# In-cell structural insight into the stability of sperm microtubule doublet

**DOI:** 10.1038/s41421-023-00606-3

**Published:** 2023-11-21

**Authors:** Linhua Tai, Guoliang Yin, Xiaojun Huang, Fei Sun, Yun Zhu

**Affiliations:** 1 National Laboratory of Biomacromolecules, CAS Center for Excellence in Biomacromolecules, Institute of Biophysics, Chinese Academy of Sciences, Beijing, China; 2 University of Chinese Academy of Sciences, Beijing, China; 3 Center for Biological Imaging, Institute of Biophysics, Chinese Academy of Sciences, Beijing, China; 4 Bioland Laboratory (Guangzhou Regenerative Medicine and Health Guangdong Laboratory), Guangzhou, Guangdong China

**Keywords:** Cryoelectron tomography, Cilia

## Abstract

The propulsion for mammalian sperm swimming is generated by flagella beating. Microtubule doublets (DMTs) along with microtubule inner proteins (MIPs) are essential structural blocks of flagella. However, the intricate molecular architecture of intact sperm DMT remains elusive. Here, by in situ cryo-electron tomography, we solved the in-cell structure of mouse sperm DMT at 4.5–7.5 Å resolutions, and built its model with 36 kinds of MIPs in 48 nm periodicity. We identified multiple copies of Tektin5 that reinforce Tektin bundle, and multiple MIPs with different periodicities that anchor the Tektin bundle to tubulin wall. This architecture contributes to a superior stability of A-tubule than B-tubule of DMT, which was revealed by structural comparison of DMTs from the intact and deformed axonemes. Our work provides an overall molecular picture of intact sperm DMT in 48 nm periodicity that is essential to understand the molecular mechanism of sperm motility as well as the related ciliopathies.

## Introduction

Sperm is a type of cell with specialized function and morphology. It carries the male partner’s genetic material and proteins to find and fuse with the female oocyte at fertilization^1^. Mature mammalian sperm consists of two main parts: a head with nucleus and acrosome, and a long tail for movement. The tail of sperm is also known as motile cilia or flagella^2^, and its core component is the axoneme, a microtubule-based super-large molecular machinery^3^. Inside the sperm axoneme, a pair of microtubule singlets termed as central pair complex (CPC) are surrounded by 9 microtubule doublets (DMTs), forming a “9 + 2” architecture^4,5^. There are hundreds of different proteins decorated on the microtubule-based structures to form a variety of functional components, such as microtubule inner proteins (MIPs), inner and outer dynein arms (IDA and ODA), radial spokes (RS), and nexin-dynein regulatory complex (N-DRC)^2^. The dynein motors are responsible for driving sperm movement and fine-tuning motility, while the MIPs provide the major stabilizing force for the DMT architecture^6^.

Significant advancements have been made in the structural studies of the sperm axoneme and DMTs. Initial discoveries revealed that the sperm axoneme has a periodicity of multiples of 8 nm with MIPs attached to the inner surface of DMTs by cryo-electron tomography (cryo-ET) and sub-tomogram averaging (STA) techniques^7–9^. Subsequent studies of cilia from different species showed that having MIPs in DMT is a common feature across different life forms, but the forms of these MIPs can vary between species^10–13^. Recent researches have provided detailed structures of purified DMTs from different sources using cryo-electron microscopy (cryo-EM) and single particle analysis (SPA) techniques^14,15^. These studies have unveiled a diverse range of MIPs and other structural features. For instance, the analysis of DMT structure from bovine trachea has demonstrated the presence of conserved MIPs shared with a species of green algae, along with a unique Tektin bundle in the A-tubule lumen^15^. Similarly, investigations on human trachea have revealed additional MIPs, contributing to a comprehensive understanding of the varied architectures of DMTs found in different cilia^16^.

Unlike trachea cilia, mammalian sperm rely on vigorous flagella beatings to overcome obstacles in the female reproductive tract. Therefore, the sperm adopt several specializations to accomplish this extreme task, such as the long axoneme, spiral-shaped mitochondria in its power-generating tail^17^, and a panoply of MIPs inside the axonemal DMT providing the stabilized force^18–21^. It showed that overall MIP patterns in sperm and trachea differ significantly within or between species^22^. The major differences are within the A-tubule lumen, where densities in the cryo-EM map of DMT in sperm axoneme are denser than trachea cilia, suggesting their different roles according to different cell motility requirements^22^. However, the details of in situ high-resolution assembly of axoneme in mammalian sperm have yet to be revealed, limiting our understanding of the structural basis of DMT in sperm beating, and the DMT-related cause of male infertility.

In this study, by using an integrative method of cryo-focused ion beam (cryo-FIB) milling, cryo-ET and STA, artificial intelligence (AI)-assisted modeling, and mass spectrometry (MS), we resolved the in-cell structure of mouse sperm axoneme DMT, achieving an overall resolution of 4.5–6.5 Å in 16 nm repeats and 6.5–7.5 Å in 48 nm repeats, and built the 16 nm and 48 nm repeat models. Then, we were able to identify 36 different MIPs in the intact mouse sperm DMT that play the roles in reinforcing the DMT lumen, giving an insight of how mammalian MIPs weave a strong interaction network to endow the structural stability of DMT. In addition, we resolved another in-cell structure of DMT from the deformed sperm axoneme and structural comparison with the intact sperm axoneme showed a superior stability of A-tubule than B-tubule in DMT. These findings not only reveal the specialized DMT structure of mammalian sperm, but also provide a structural basis for the treatment of human infertility caused by DMT defects.

## Results

### In-cell molecular architecture of mouse sperm axoneme DMT

While multiple studies have explored structure of axoneme DMT^14,15^, the majority of these investigations relied on isolated DMT samples. Unfortunately, this approach compromised the integrity of the axoneme’s natural structure. In light of this, our study opted to directly freeze mouse sperm for cryo-ET and STA, aiming to examine their in situ structure at a high resolution. However, the thickness of the mouse sperm tail (> 300 nm) posed a challenge for high-resolution cryo-ET. To overcome this obstacle, we employed cryo-FIB to thin the sample prior to data collection.

After extracting the mouse sperm directly from the testis and vitrification immediately, we employed two different sample preparation approaches and collected cryo-ET datasets separately. In the first approach, sperm were vitrified on the grid with large holes (R 3.5/1) to preserve the natural state of axoneme, followed by cryo-FIB milling to prepare the cryo-lamellae (Supplementary Fig. S1), which were used for the subsequent cryo-ET data collection (F-dataset). In the second approach, sperm were vitrified on the grid with smaller holes (R 1.2/1/3), and then the whole axoneme was used directly for cryo-ET data collection (W-dataset) at the region of sperm principal piece. In line with previous investigations of cilia showing multiple periodicities of DMTs^14–16,23^, we solved three structural models of mouse sperm axonemal DMTs in the F-dataset (Supplementary Table S1) with the periodicities of 16 nm (DMT_F16_), 48 nm (DMT_F48_), and 96 nm (DMT_F96_), respectively. For the W-dataset (Supplementary Table S1), we solved two structural models with the periodicities of 16 nm (DMT_W16_) and 48 nm (DMT_W48_), respectively.

**Fig. 1:**
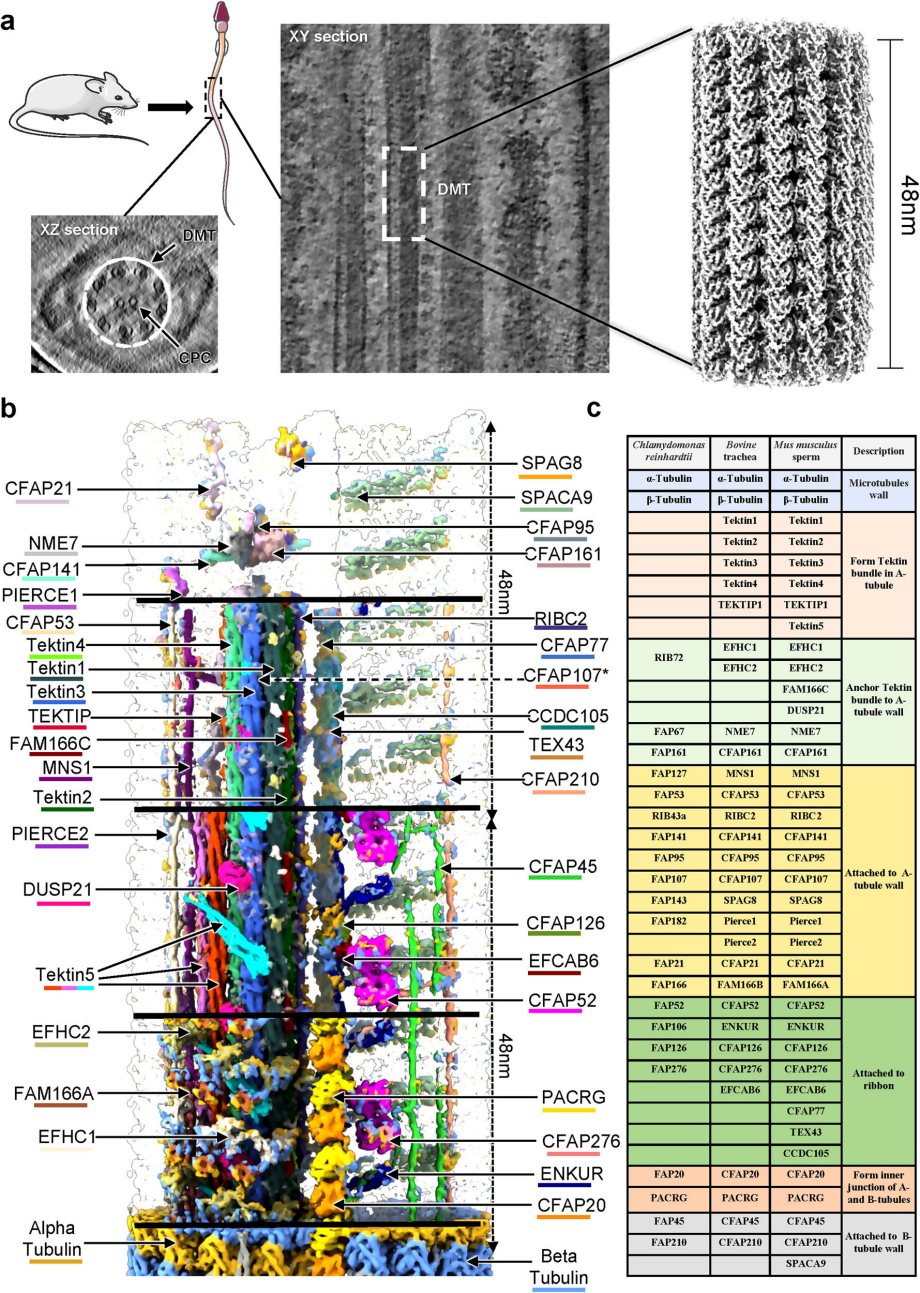
Overall architecture of mouse sperm DMT. **a** Schematic representation of in-cell structural determination of mouse sperm DMT. The transverse (**a**) and vertical (**b**) sections of mouse sperm axoneme are shown as the indicated slices in cryo-electron tomograms, respectively. CPC, central pair complex. DMT, microtubule doublet. A high resolution cryo-EM map of DMT with a spatial periodicity of 48 nm was obtained by sub-tomogram analysis. **b** Molecular dissection of DMT architecture showing with two 48 nm repeats of DMT cryo-EM map. The layers of the DMT structure were sequentially removed from bottom to top to reveal its inner composition. The tubulin subunits as well as MIP subunits are colored and labeled individually. The color schemes are summarized in Supplementary Table S5. To be noted, the subunit CFAP107 is hidden by other MIPs and could not be shown in the current view. **c** List of names and functions of tubulins and MIPs from three representative specimen, *Chlamydomonas reinhardtii*, *Bovine* trachea, and *Mus musculus* sperm. Different colors represent different functional roles of MIPs in the architecture of DMT.

In the F-dataset, the circular “9 + 2” architecture of the sperm axoneme can be well observed from the cross sections of selected tomograms of proper cryo-lamellae that could just hold an intact axoneme (Fig. 1a), suggesting an intact state of sperm axoneme preserved after vitrification. Although many tomograms do not contain a complete axoneme due to the limited thickness of cryo-lamellae by cryo-FIB milling^24–26^, major components of the axoneme can be clearly observed (Fig. 1a), allowing manual picking of DMT particles in a consistent direction. To push a higher resolution, different local masks focused on A- and B-tubules were employed during image processing (Supplementary Fig. S2). The A-tubule was resolved at an overall resolution of 4.5 Å with the local resolution up to 3.6 Å in DMT_F16_, and an overall resolution of ~6.5 Å in DMT_F48_ (Supplementary Figs. S3,S4). The B-tubule was resolved at an overall resolution of 6.5 Å in DMT_F16_ and 7.5 Å in DMT_F48_ (Supplementary Figs. S3,S4). Based on the map of DMT_F96_, we confirmed that mouse sperm axoneme DMT has an overall periodicity of 48 nm (Supplementary Fig. S5), consistent with the previous studies of cilia DMTs^14,15^. For the W-dataset, similar image processing strategies were applied (Supplementary Fig. S6), resulting in maps of DMT_W16_ and DMT_W48_ with the resolutions of 7.9 Å and 8.6 Å, respectively (Supplementary Fig. S7).

**Fig. 2:**
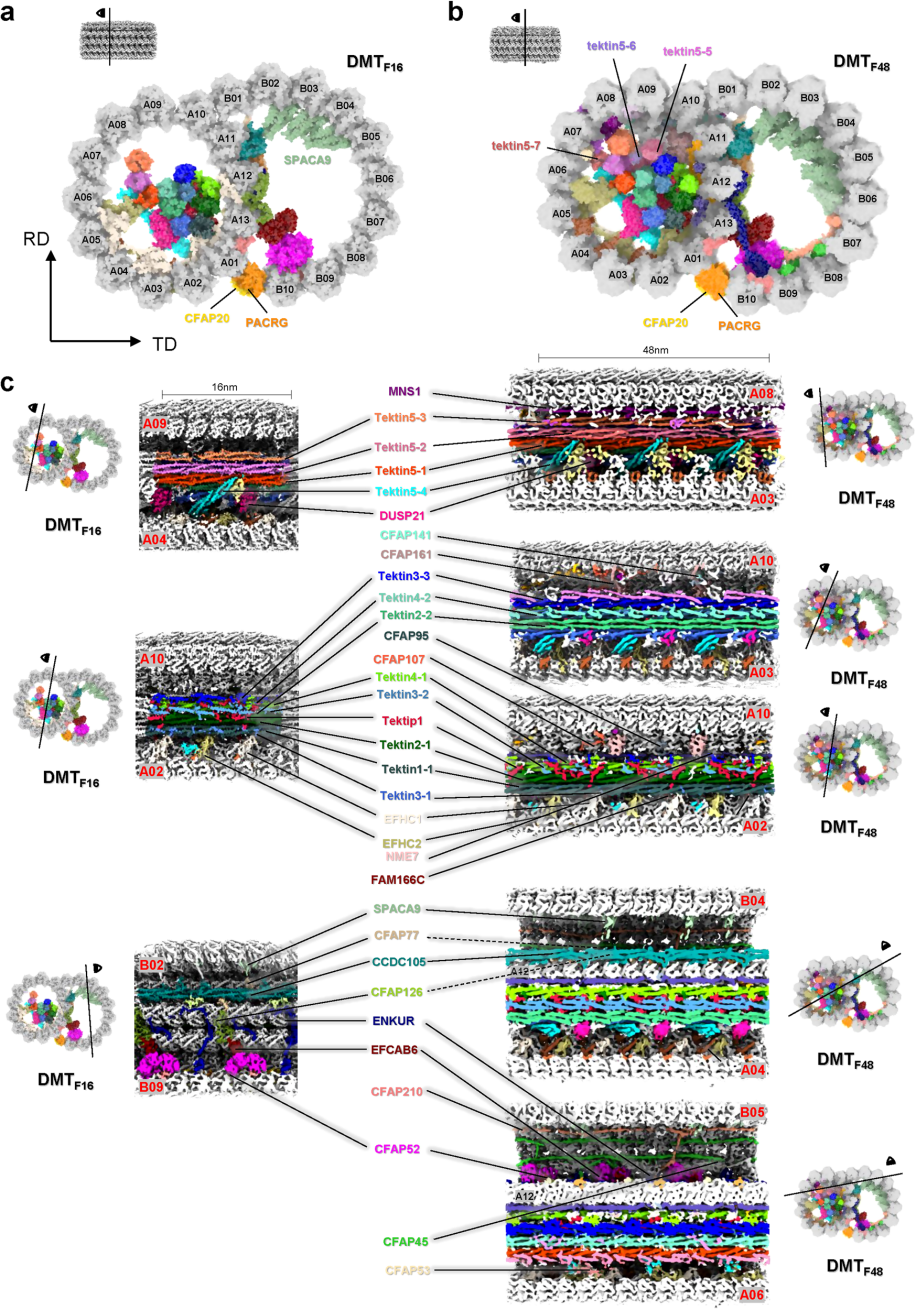
Detailed molecular organization of DMT in the repeats of 16 nm and 48 nm cryo-EM maps and models. **a** One representative transverse section of 16 nm repeat TDMT_F16_ model. TD, tangent direction; RD, radial direction. Figure was generated by 3D PROTEIN IMAGING server^74^. **b** One representative transverse section of 48 nm repeat DMT_F48_ model. The tubulins are numbered and MIPs are colored individually in both A- and B-tubules with CFAP20 and PACRG (**a**, **b**) and Tektin5 proteins labeled (**b**). Figure was generated by 3D PROTEIN IMAGING server^74^. **c** Serials of representative vertical sections of DMT cryo-EM maps in both 16 nm and 48 nm repeats. The position of each vertical section is described aside. The MIPs in both A- and B-tubules are colored and labeled individually. The color schemes are summarized in Supplementary Table S5.

In these maps, it is not difficult to see that A-tubule has a rich protein density, which has also been reported in previous studies^27^. Owing to the resolution limitation, it is not feasible to accurately determine the protein types or isotypes solely from the cryo-ET data, even in the case of tubulins exhibiting relatively high local resolutions. To identify multiple MIPs in the DMT map, we analyzed the proteome of mouse sperm using mass spectrometry (MS) (Supplementary Fig. S8 and Table S2). Subsequently, we integrated the MS data with the structural study of axoneme DMT in homologous species^14,28^, and AI-based protein structure prediction approach^29,30^ to build the model of mouse sperm axonemal DMT from DMT_F16_ and DMT_F48_ maps (Figs. 1b, c and 2a, b, see also “Materials and methods”). At the high-resolution region of DMT_F16_ map, well-defined side-chain densities of distinguishable residues facilitated accurate assignment and modeling of multiple MIPs (Supplementary Figs. S9–S11). For MIPs in middle-resolution area of DMT_F16_ and DMT_F48_ maps, the secondary structural elements can be resolved and the assignment and modeling of MIPs were performed with the aid by AI structural predictions and the reference by previously reported MIP structures from bovine trachea (Supplementary Fig. S12)^15^. We used findMySequence^30^ to identify the most possible candidate sequence of each MIP based on the complete mouse genome, yielding the successful assignments of 18 MIPs in the DMT_F16_ map with high confidence scores (Supplementary Tables S3,S4). For those MIPs not assigned by findMySequence, we started structural predictions by AlphaFold2^29^, refined the models based on the map and verified the model-to-map fitting quality (Supplementary Figs. S13,S14). For FAM166C, TEX43 and CFAP77, they could only be placed by referring to the preprint report on the composition of bovine sperm axoneme^28^.

In addition to α- and β-tubulins, we identified and modeled 36 different kinds of MIPs in our intact mouse sperm DMT reconstruction (Figs. 1b-c, 2c; Supplementary Videos S1-S2). Among these, 31 MIPs were previously observed in tracheal DMT structures^15^, while the remaining five MIPs were sperm-specific MIPs that highly expressed in testis tissue^31^, including Tektin5, CCDC105, DUSP21, CFAP77 and TEX43. To be specific, in addition to the previously reported Tektin proteins, Tektin1, Tektin2, Tektin3, Tektin4 and Tektip1 in trachea cilia, we identified and modeled a new Tektin protein Tektin5 that is predominantly expressed in sperm^32–34^. Besides, we modeled 6 MIPs (EFHC1, EFHC2, FAM166C, DUSP21, NME7 and CFAP161) that anchor Tektin bundle to A-tubule wall. The additional MIPs that may play the role of stabilizing A-tubule wall include MNS1, CFAP53, RIBC2, CFAP141, CFAP95, CFAP107, SPAG8, Pierce1, Pierce2, CFAP21 and FAM166A. The modeled MIPs to attach and may stabilize the ribbon (defined by the protofilaments A11, A12 and A13) of DMT comprise CFAP52, ENKUR, CFAP126, CFAP276, EFCAB6, CFAP77, TEX43 and CCDC105. The junction MIPs (CFAP20 and PACRG) between A and B-tubules as well as the MIPs (CFAP45, CFAP120 and SPACA9) attaching the inner surface of B-tubule are also well modeled, where SPACA9 was not modeled in the bovine trachea cilia^15^.

Overall, the architecture of intact mouse sperm axonemal DMT is consistent with the previous low resolution (> 1 nm) study^35^ but shows significant differences in comparison with the structure of DMT from trachea cilia. Compared to the previously reported DMT structures from bovine tracheal and human trachea, mouse sperm axonemal DMT structure not only exhibits an overall conserved architecture with many conserved subunits but also shows specific detailed features with many new components (Supplementary Fig. S15), indicating intrinsic structural variations between sperm axoneme and trachea cilia^14–16,23^.

### The extended tektin bundle in A-tubule

**Fig. 3:**
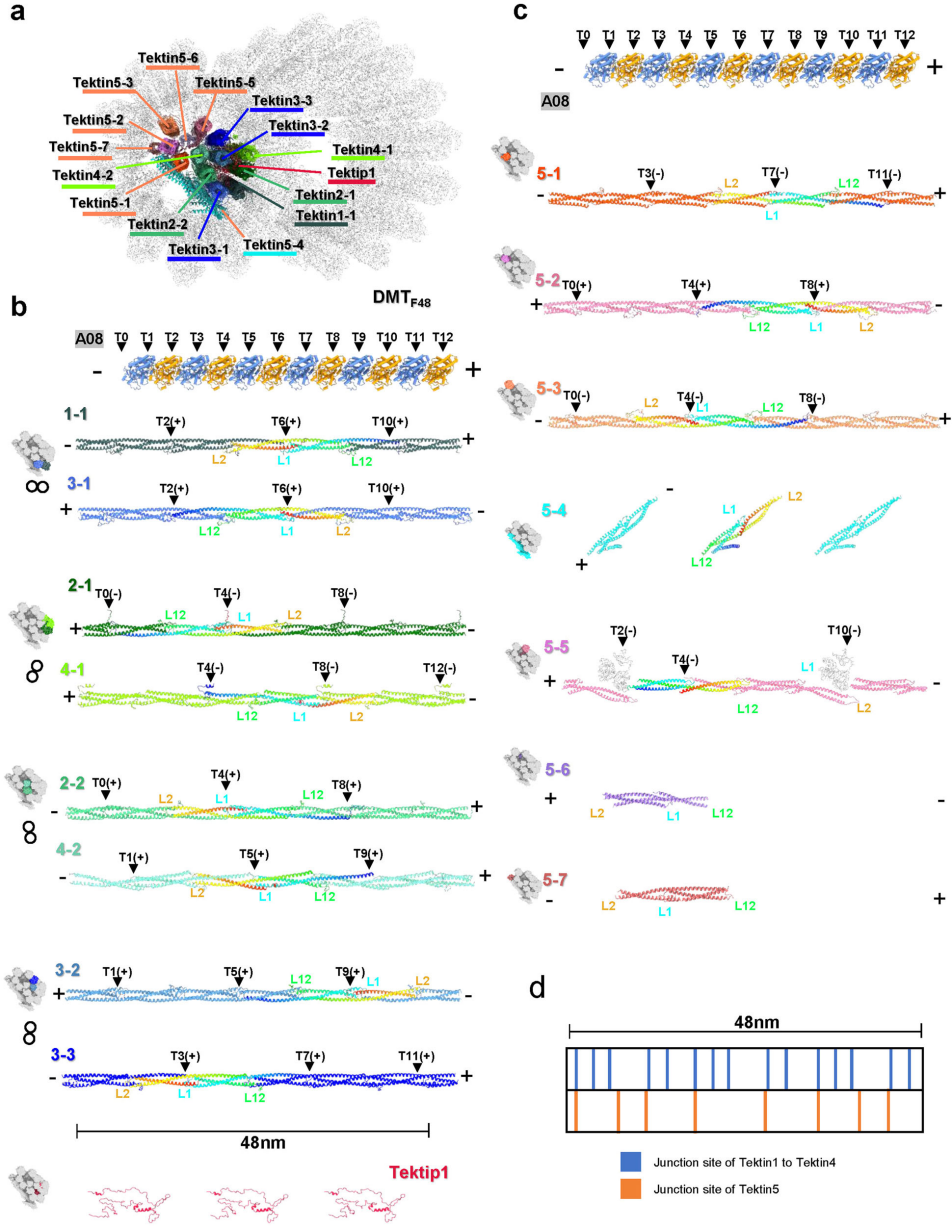
Assembly of Tektin proteins in sperm axoneme DMT. **a** A transverse section view of DMT_F48_ model to show the Tektin bundle in the A-tubule with Tektin proteins labeled. The coloring schemes are summarized in Supplementary Table S5. **b** Parallel comparisons of four pairs of Tektin1/2/3/4 filaments in each 48 nm repeat. The relative position of each pair in the Tektin1/2/3/4 bundle is shown and colored aside left. The sequential tubulins of the protofilament A08 form a 48 nm axis along the sperm axoneme with the coordinates from T0 to T12 and the direction from minus-end to plus-end of microtubule. The direction of Tektin filament is defined as the one from the C-terminus to N-terminus of one Tektin protein. The junction sites of Tektin filaments are indicated with black triangles and labeled with the coordinates Tx(+/−). Tx(+) means the site locates right to Tx and Tx(−) means left to Tx. **c** Parallel comparisons of Tektin5 proteins in each 48 nm repeat along with the axis defined by tubulins and with the junction sites labeled accordingly. Their positions in the Tektin bundle are also shown and colored aside left. **d** The distribution of junction sites of Tektin proteins in each 48 nm repeat.

The Tektin family of proteins (Tektin1/2/3/4/5) play the crucial role for the formation of the axoneme. Previous biochemical and knockout experiments have indicated that Tektin2, Tektin3, and Tektin4 may be genes associated with asthenospermia (Fig. 3a)^36–38^. The absence of Tektin3 and Tektin4 leads to a decrease in sperm motility^36,37^. In our structure, the Tektin protein possesses a triple helix bundle in its main body (Supplementary Fig. S16a), and assembles into a long filament via the canonical head-to-tail interactions (Fig. 3b). These coiled-coil patterns along the DMT align with previous findings^39–42^. Notably, Tektin1/2/3/4 have been observed to form an 8-membered Tektin bundle within the A-tubule of DMT in bovine trachea cilia, contributing to overall structural stability^15,36,37,43,44^. Interestingly, Tektin5 is expressed exclusively in the testis and is localized throughout the sperm tail^45^. Although Tektin5 was suggested to be involved in the formation of the extensive helix bundle inside the sperm axoneme DMT A-tubule^46^, its exact location and copy number was not clear in the relative low resolution cryo-ET maps of previous studies^22,35^.

Based on our higher resolution in-cell DMT_F16_ map with the side chain density of many distinguishable residues, we identified and modeled the Tektin proteins inside the mouse sperm axoneme DMT. In the case of Tektin1/2/3/4 proteins, they exhibit comparable organizational principles to those observed in bovine trachea DMT^15^. Specifically, four pairs of these fibers come together to form an 8-Tektin bundle, with Tektip1 serving as the central hub for interactions (Fig. 3b).

Interestingly, Tektin5 seems to form an additional bundle itself (Fig. 3a, c). Domain distribution of full-length Tektin5 is basically the same as Tektin1 to Tektin4, comprising two long helices called 1 A and 2 A, two short helices called 1B and 2B, three loops called L1, L12, and L2 separated by these helices (Supplementary Fig. S16a)^15^. Unlike Tektin1 to Tektin4 that share essentially the same tertiary architecture (Supplementary Fig. S16b), Tektin5 proteins adopt variant conformations (Supplementary Fig. S16c, d). Four groups of Tektin5 with the periodicity of 16 nm were identified in DMT_F16_ map, named Tektin5-1, Tektin5-2, Tektin5-3 and Tektin5-4. Among them, Tektin5-1/2/3 form a continuous homopolymer filament through the typical head-to-tail interactions (Fig. 3c). Yet Tektin5-4 adopts a completely different configuration. Instead of forming a homopolymer filament parallel to the Tektin bundle, Tektin5-4 monomer tilts about 45 degrees and lies on the outside of Tektin bundle (Fig. 3a). Each Tektin5-4 monomer is shorter than the normal Tektin protein because its N-terminal helix folds back to form a four-helix bundle (Supplementary Fig. S16c).

More interestingly, in DMT_F48_ map, we built three additional groups of Tektin5 proteins, named Tektin5-5, Tektin5-6 and Tektin5-7. There are three Tektin5-5 proteins with one in form a and two in form b (Supplementary Fig. S16c) in each 48 nm repeat (Fig. 3c). Form a and form b interact with each other in a head-to-tail way and connect indirectly with the third Tekin5-5 protein (form b) via the interactions with two NME7 proteins (Fig. 3c, see also the next section). The presence of NME7 forms a severe steric hindrance of Tektin5-5 filament, inducing a significant bending of Tektin5-5 in form b at the L2-loop (Fig. 3c). For Tektin5-6 and Tektin5-7, there is only one copy in each 48 nm repeat, respectively (Fig. 3c), locating at the opposite position of Tektin5-2 filament (Fig. 3a). Considering that we assigned the proteins of Tektin5-3/4/5/6/7 in the model based on its higher abundance than other Tektin isotypes in MS, these structural characteristics require further validation by higher resolution structural evidence.

When we compared these Tektin filaments side by side along with the tubulin wall, we found their directions and junction sites are interleaved. We selected 12 sequential tubulins of the protofilament A08 to make a 48 nm axis along the sperm axoneme with the coordinates from T0 to T12, and the direction from minus-end (–) to plus-end (+) of microtubule. We further defined the direction of Tektin filament as the one from the C-terminus (–) to N-terminus (+) of Tektin protein. Then we found that, for the eight-membered helix bundle core formed by Tektin1/2/3/4, half of the filaments (Tektin1-1, Tektin2-2, Tektin4-2, and Tektin3-3) have the positive direction and another half (Tektin3-1, Tektin2-1, Tektin4-1, and Tektin3-2) with the negative direction. And their junction sites are arranged in an offset manner, following the direction of the tubulin protofilament, e.g., Tektin1-1 has the junction sites at T2, T6, and T10 while Tektin2-1 has the junction sites at T0, T4, and T8 (Fig. 3b). This specific arrangement of Tektin filaments may reinforce the tubulin wall in both directions and in multiple positions (Fig. 3d). The 7 groups of Tektin5 proteins are also arranged in a similar way. Tektin5-1, 5-3, and 5-7 orient with the positive direction, while Tektin5-2, 5-5, and 5-6 orient with the negative direction. Specially, Tektin5-4 proteins make a tilted direction. The junction sites of Tektin5 filaments are dispersedly distributed in each 48 nm repeat but interleaved with the junction sites of Tektin1 to Tektin4 filaments (Fig. 3d), which may further stabilize the DMT architecture.

### Intricate interaction network of MTIPs in A-tubule

The multiple MIPs in the A-tubule are Tbound together by a rich interaction network, which can be divided into two groups, the interactions within the Tektin bundles and the ones between the Tektin bundles and the tubulin wall. For the interactions within the Tektin bundles (Supplementary Fig. S17), the entire Tektin5 bundle anchors Tektin1/2/3/4 bundle mainly by the interactions between Tektin5-1 and Tektin4-2 (Supplementary Fig. S17a). Inside the Tektin5 bundle, we observed the interactions between Tektin5-1 and Tektin5-2 (Supplementary Fig. S17b), and between Tektin5-2 and Tektin5-3 (Supplementary Fig. S17c). Besides, inside the Tektin1/2/3/4 bundle, there are interactions between Tektip1 and Tektin2-1, 2-2, 3-1, 3-2, 3-3 and 4-1, respectively (Supplementary Fig. S17d–i), which are conserved in comparison with the interactions within Tektin1/2/3/4 bundle of bovine tracheal cilia^15^.

**Fig. 4:**
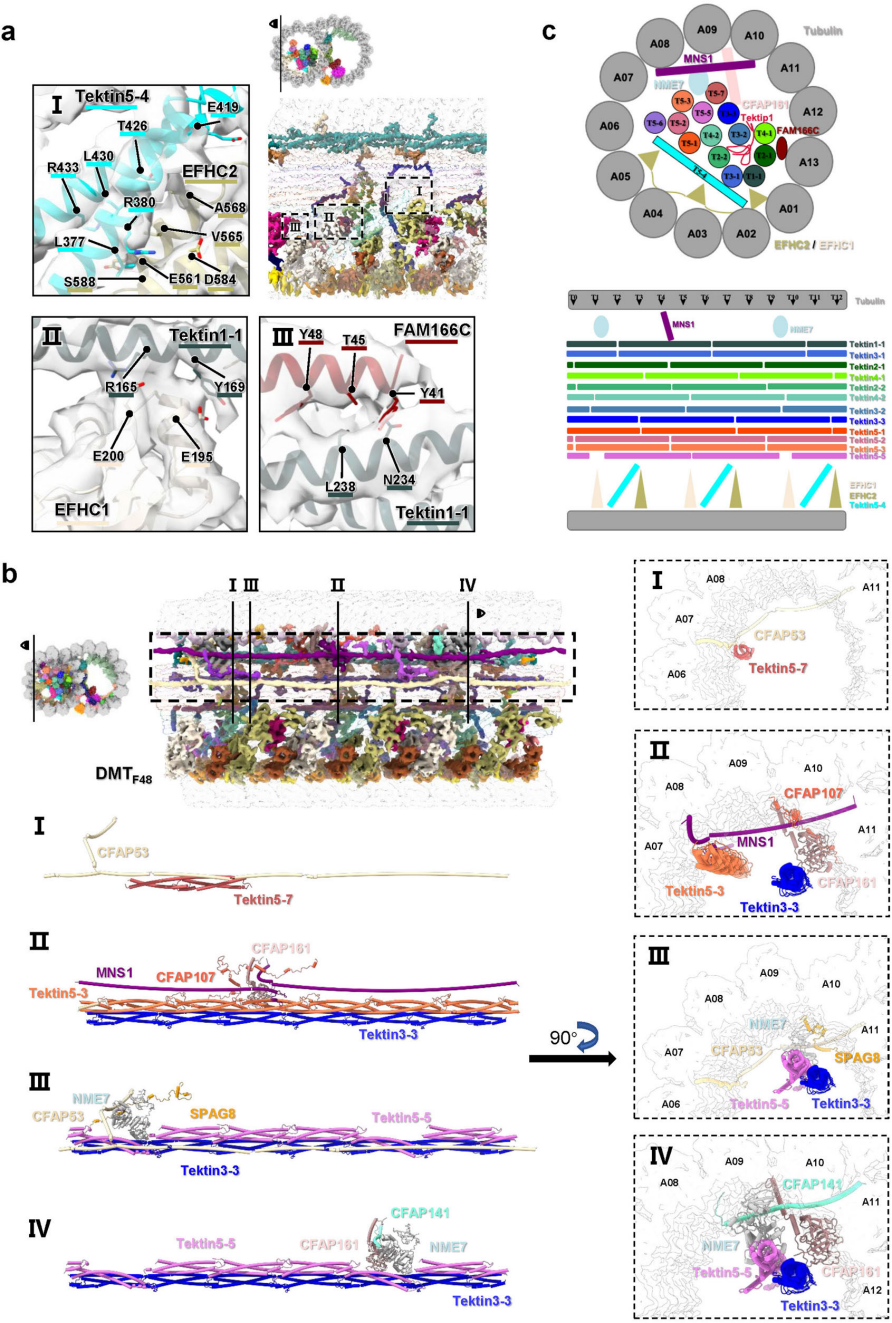
Interactions between MIPs and Tektin bundles in A-tubule and between MIPs in B-tubule. **a** Interactions with 16 nm periodicity. The vertical sections of DMT_F16_ map at the A-tubule and B-tubule are shown, respectively, and the MIPs responsible for anchoring Tektin bundles onto the tubulin wall in A-tubule, as well as the MIPs interacting with the ribbons in B-tubule, are highlighted and colored accordingly (Supplementary Table S5). The Tektin bundles and tubulin wall are shown in low light and transparency. The interaction interfaces with residues labeled between Tektin5-4 and EFHC2, Tektin1-1 and EFHC1, Tektin1-1 and FAM166C in A-tubule are zoomed in and shown in Panel I-III, respectively. All contacting residues were recognized using default parameters in UCSF ChimeraX^48^. **b** Interactions with 48 nm periodicity. The vertical sections of DMT_F48_ map at the A-tubule is shown and the MIPs responsible for anchoring Tektin bundle onto the tubulin wall are highlighted and colored accordingly (Supplementary Table S5). The Tektin bundles and tubulin wall are hidden. The interactions between Tektin5-7 and CFAP53, among Tektin5-3 filament, Tektin3-3 filament, MNS1, CFAP107, and CFAP161, among Tektin5-5 filament, Tektin3-3 filament, SPAG8, NME7, and CFAP53, and among Tektin5-5 filament, Tektin3-3 filament, CFAP141, and NME7, are zoomed in and highlighted in Panel I–IV with vertical and transverse section views, respectively. **c** A schematic model of MIPs interactions in A-tubule.

The interactions between the Tektin bundles and the tubulin wall are mainly mediated by multiple MIPs including EFHC1, EFHC2, FAM166C, CFAP53, CFAP141, CFAP161, NME7, SPAG8 etc., which play the roles of anchoring Tektin bundles onto the tubulin wall, providing strength along with or perpendicular to the A-tubule (Fig. 4). It was revealed that in bovine trachea cilia EFHC1, EFHC2, RIBC2, CFAP161, NME7, and also tubulin protofilament A12 are responsible for the attachment of Tektin bundles to A-tubule wall^15^. In our DMT structure of intact mouse sperm axoneme, for each 16 nm repeat, we observed the interlocked interactions between the C-terminal region of Tektin5-4 and EFHC2, between Tektin1-1 and EFHC1, and between Tektin1-1 and the N-terminal region of FAM166C (Fig. 4a). To be noted, FAM166C was reported present in human trachea cilia with the role of anchoring Tektin1-1 onto the tubulin wall^16^. For each 48 nm repeat, we observed more interlocked interactions at various transverse sections (Fig. 4b). The helical protein CFAP53 interacts with Tektin5-7 in parallel by using its second helix on one side and interacts with the tubulin protofilaments A06-A07 on the other side. Another helical protein MNS1 lies between tubulin protofilaments A07-A08 and interacts with Tektin5-3 at its C-terminal region. The junction site of two MNS1 proteins is interlocked by interacting with CFAP107 and CFAP151 that interacts with Tektin3-3. The junction sites of Tektin5-5 filaments are interlocked by the complexes of NME7/CFAP53/SPAG8 and NME7/CFAP141/CFAP161, respectively. These two complexes anchor Tektin5-5/Tektin3-3 filaments onto the tubulin wall at the sites of A07/A11 and A08/A11/A12, respectively. We also noted that the presence of NME7 with the periodicity of 48 nm hinders Tektin5-5 proteins to assemble into a long filament as Tektin5-1 to Tektin5-3, resulting in the 48 nm periodicity of Tektin5-5 filament (see also Fig. 3c).

With the above structural analyses, we were able to create a schematic diagram of MIPs organization within A-tubule (Fig. 4c). When viewing the junction sites of Tektin bundle, different Tektin filaments line up in an offset fashion to ensure that their junction sites are evenly distributed along the vertical axis. This kind of architecture may prevent the Tektin bundle from tearing apart when the external stress is applied to A-tubule, just like a strictly rebar-like appearance. The Tektin bundle is linked to the tubulin wall by several buffer proteins, like EFHC1/EFHC2, MNS1 and NME7 with different periodicities, to reinforce the stability of tubulin wall. From the view of transverse section (Fig. 4c), the tilted Tektin5-5 provides additional supporting force for the Tektin bundle, EFHC1 and EFHC2 reinforce the protofilaments A01 to A05, FAM166C for the protofilament A13, and MNS1 for the protofilaments A08 to A10. As a result, MIPs in A-tubule are efficiently assembled and work together to reinforce the structural stability of DMT.

### Organization and interaction of MIPs in B-tubule

**Fig. 5:**
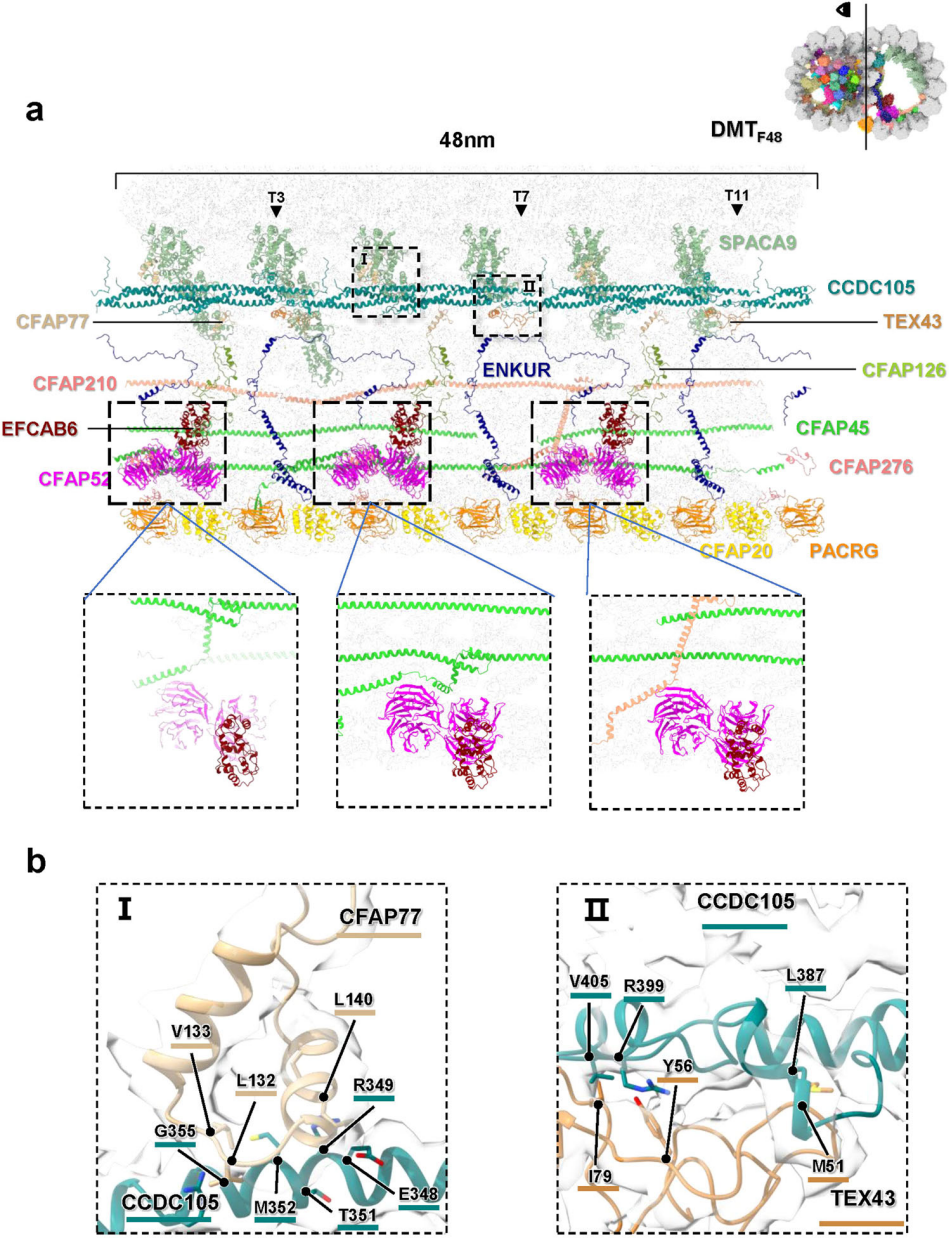
Structure organization of MIPs in B-tubule. **a** Structural models of all MIPs in the B-tubule are shown with a 48 nm length of vertical section nearby the ribbon region, labeled and colored accordingly (Supplementary Table S5). The coordinates (T3, T7 and T11) of junction sites of CCDC105 filament are indicated. The interactions between CFAP52/EFCAB6 in 16 nm repeat and CFAP45/CFAP276 in 48 nm repeat are zoomed in. **b** The interaction interfaces between CCDC105 and CFAP77, and CCDC105 and TEX43 in B-tubule, which are indicated in **a**, are zoomed in and shown in Panel I and II with residues labeled, respectively.

The B-tubule is formed by the protofilaments B01 to B10, enveloping the ribbon region that encompasses protofilaments A11 to A13 (Fig. 2a, b). The interstice between protofilaments A01 and B10 is linked by the inner junction, which is composed of PACRG and CFAP20 (Figs. 1b, 2a, and 5a). These proteins, PACRG and CFAP20, exhibit a remarkable conservation across eukaryotes^14–16,47^. Compared to the highly decorated A-tubule, B-tubule possess less MIPs, including CCDC105, TEX43, CFAP77, CFAP126, CFAP276, EFCAB6, CFAP52 and ENKUR (Fig. 5a), which attach to the ribbon region and extend into the B-tubule lumen. Additional MIPs, namely SPACA9, CFAP45, and CFAP210, decorate the inner wall (B02-B10) of B-tubule (Fig. 5a and Supplementary Fig. S9).

As one of the largest proteins attached to the ribbon, CCDC105 comprises a three-helix bundle in its main body with an additional helix at its N-terminus (Supplementary Fig. S16e). Structural superposition reveals CCDC105 resembles a similar domain configuration with Tektin proteins and the r.m.s.d. of C-alpha atoms between CCDC105 and Tektin4-1 is 1.35 Å that is calculated by UCSF ChimeraX^48^. CCDC105 resides between the protofilaments A11 and A12, assembling into a long head-to-tail filament by interacting with the neighbor CCDC105 molecules. The coordinates of junction sites of CCDC105 filament are T3, T7 and T11, respectively (Fig. 5a). At the junction site, we observed that there is a small protein TEX43 clamping the L12 loop (L387-R399) of CCDC105 (Fig. 5a, b). It also interacts with the tubulin wall at the A12 protofilament, acting as a rivet to strength the junction site of CCDC105 filament (Fig. 5a). Another small protein CFAP77 also interacts with CCDC105 at the region of M352-G355 to stabilize the ribbon (Fig. 5b).

ENKUR that has an extremely elongated configuration (Fig. 5a) connects the protofilaments A12, A13, and B10 in every 16 nm repeat, and participates in the inter-protofilament interactions of B-tubule. We observed a potential interaction between the middle region of ENKUR and the loop region of TEX43, suggesting a role of reinforcement.

EFCAB6, the MIP with no ortholog in *Chlamydomonas*, was reported to attach to the protofilament A13 in the B-tubule of bovine trachea cilia DMT with 48 nm periodicity^15^. Interestingly, in our structure of intact mouse sperm axonemal DMT, we found EFCAB6 with the periodicity of 16 nm, each EFCAB6 interacts with CFAP52, which contains two WD40 domains and attaches to the CFAP45 or CFAP210 filaments (Fig. 5a). The additional copies of EFCAB6/CFAP52 pairs provide a stronger support to the protofilaments A13 and B10, in comparison with that of trachea cilia^15,49^ (Fig. 5a).

Both CFAP45 and CFAP210 proteins exhibit a 48 nm periodicity and possess long helices to form filaments attaching to the protofilaments B07-B09 (Figs. 2b, c and 5a)^49^. The two copies of CFAP45, situated on protofilaments B08 and B09 in parallel, adopt different tertiary configurations in the N-terminus. The N-terminal helix in one CFAP45 contains an elbow bend to accommodate the steric hindrance of CFAP52 (Fig. 5a), and the same helix in another CFAP45 protein are plain helices. CFAP210 also contains an elbow bend at its N-terminal helix across protofilaments B07 to B09 to accommodate the steric hindrance of CFAP52 (Fig. 5a), while its rest part lies between protofilaments B06 and B07.

The most outstanding features of B-tubule are the pyramid-shaped densities decorated on its inner surface at the region of protofilaments B02 to B06 (Fig. 2b). Both recent studies on human sperm microtubule singlets (SMT) and trachea DMT showed that it is SPACA9 with a pyramid-shaped structure to decorate both lumens of DMT B-tubule and SMT with the same repeat pattern^16,50^, but it was not found in purified mouse sperm DMT^51^. In the DMT of human trachea cilia, SPACA9 was reported to have an 8 nm periodicity and situated in between the α- and β-tubulin heterodimers^16^. At every 8 nm, three SPACA9 monomers are situated between the protofilaments B02 to B05, forming a hierarchy structure^16^. However, in the DMT structure of mouse sperm axoneme, we observed that SPACA9 forms a pattern of 5-3-3-4-4-4 in each 48 nm repeat, and SPACA9s are located between the protofilaments B02 to B07, B02 to B05, and B02 to B06, respectively (Fig. 5a and Supplementary Fig. S9). Since SPACA9 is located at the cross-sectional position between tubulin heterodimers and different protofilaments, these abundant SPACA9 proteins provide additional stabilization to the B-tubule walls. To be noted, although SAXO1 was found to mediate the interaction between SPACA9 and the tubulin wall in DMT of human trachea cilia^52^, we did not identify the equivalent in our structure, which might be due to the limited resolution of the local region.

### DMT structure in a deformed sperm axoneme

**Fig. 6:**
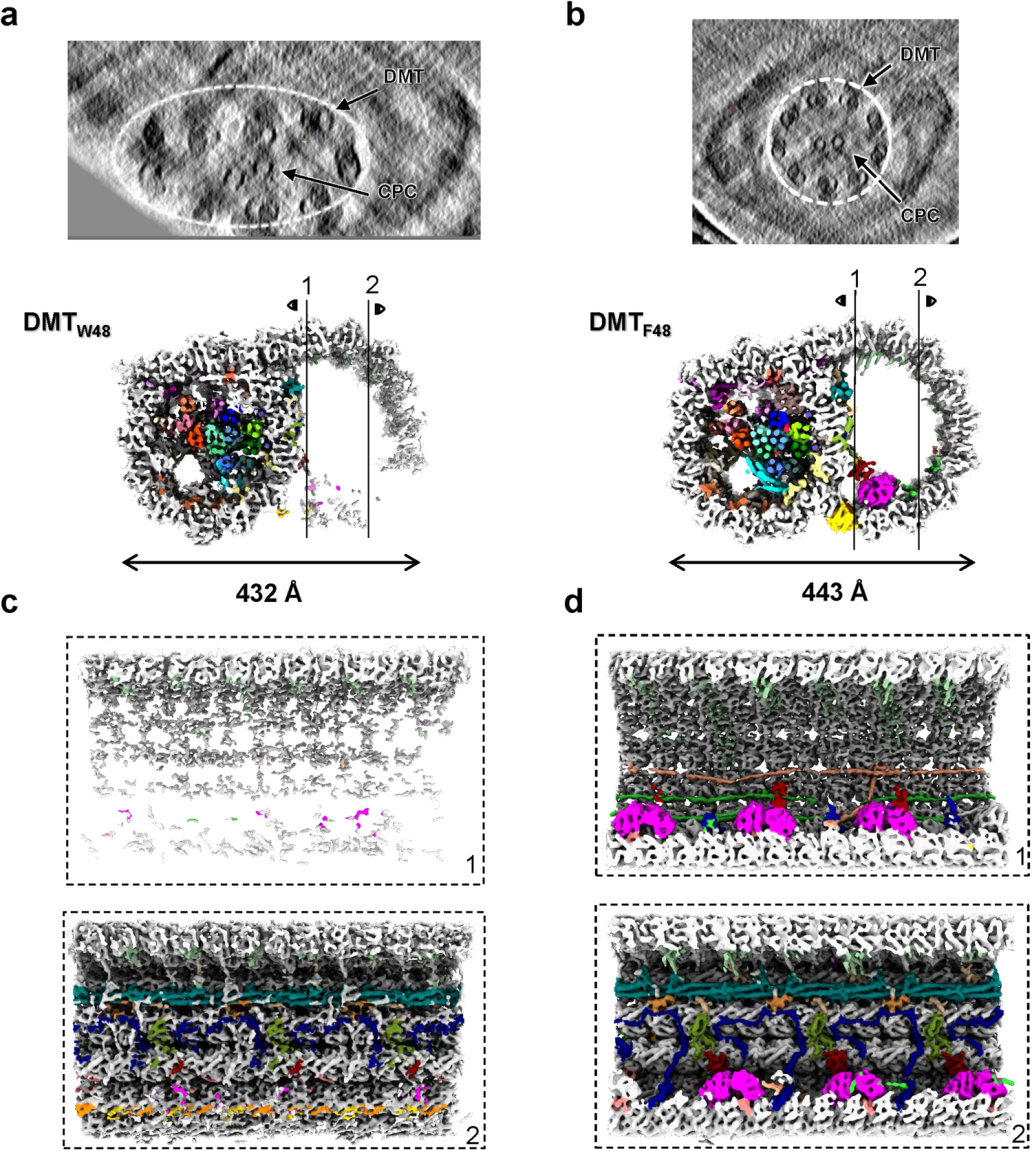
Comparison of DMT structures between deformed (DMT_W48_ map) and intact (DMT_F48_ map) axonemes of mouse sperm. **a**, **b** Selected tomograms of W-dataset (**a**) and F-dataset (**b**) are shown in XZ sections with DMT and CPC labeled. The transverse sections of DMT_W48_ (**a**) and DMT_F48_ (**b**) maps are shown with MIPs densities colored, respectively. **c**, **d** Two vertical sections of DMT_W48_ and DMT_F48_ maps are shown with MIPs densities colored, respectively. The section positions (1 and 2) are indicated in **a** and **b**, accordingly.

Since there are more MIPs in A-tubule than that in B-tubule (Supplementary Fig. S18), A-tubule would exhibit a better structural stability than B-tubule. The cryo-ET dataset (W-dataset) using the sperm specimen vitrified on the grids with a smaller hole (R1.2/1/3) resulted the tomograms with the deformed sperm axoneme (Fig. 6a), providing us an opportunity to investigate the role of MIPs in maintaining the structural stability of sperm axoneme DMT. In this dataset, the “9 + 2” architecture of the sperm axoneme is compressed along the electron illumination direction (Supplementary Fig. S19), possibly due to the surface tension applied to the sperm tail during blotting. By analyzing the cryo-EM maps of DMT_W48_ and DMT_F48_ (Fig. 6a, b), in addition to the different widths, 432 Å for DMT_W48_ and 443 Å for DMT_F48_, we further found that the A-tubule in the deform specimen (DMT_W48_) shows a similar structure as that in the intact specimen (DMT_F48_), while the densities of B-tubule in the deformed one (DMT_W48_) are significantly averaged out (Fig. 6c, d).

Comparing the DMT structures between the deformed and intact states, we observed that the 13 protofilaments of A-tubule exhibit the same structure in both datasets. In the A-tubule lumen, although the Tektin bundle between two specimens remains unaffected, we observed significant different densities in the bridging components such as EFHC1/2 and DUSP21 that are involved in anchoring the Tektin bundle to the tubulin wall (Supplementary Fig. S20). It suggests that the Tektin bundle provides the main role to support the A-tubule stability when responding the external forces, and the bridging components may disperse or buffer the external forces through appropriate conformational changes to minimize the deformation of the tubulin wall.

However, in the B-tubule, we observed a substantial loss of density in both the tubulin walls and MIPs within the DMT_W48_ map, including CFAP20, PACRG, SPACA9, and CFAP52 (Fig. 6c, d). It indicates that these proteins are the most fragile part of sperm axonemal DMT whose structures are the most vulnerable to external forces. The inner junction formed by CFAP20 and PACRG is probably the most stressed position in the deformed sperm DMT, because the density of inner junction has almost disappeared in DMT_W48_ map (Fig. 6a). The stability of the B-tubule is considerably weaker compared to that of the A-tubule, aligning with previous findings^53^. On the other side of the B-tubule, the densities of tubulin walls were preserved (Fig. 6c, d), which may be due to the interaction between A10 and B01 protofilament and the reinforcement by SPACA9 proteins.

As a result, by comparing the sperm axonemal DMT structures in the intact and deformed states, we found MIPs in DMT play different roles in maintaining the overall structures of A- and B-tubules. The different compositions of MIPs result in a great difference in the stability of A- and B-tubules. The stability of A-tubule may be due to the abundance of Tektin proteins that form Tektin bundle in the lumen. However, it is worth noting that the axoneme samples in the F- and W-datasets may not exhibit complete consistency. For instance, the axoneme in the W-dataset primarily resides in the principal piece of mouse sperm. Therefore, the structural difference of DMT between these two datasets may encompass structural specialization of DMTs along the length of the sperm flagella, which requires further investigations.

## Discussion

In this study, we solved the in-cell molecular structure of mouse sperm axonemal DMT with an overall resolution of 4.5–7.5 Å, and built 36 different MIPs within the 48 nm repeat (Supplementary Video S3). We found, in mouse sperm axonemal DMT, the lumen of A-tubule is almost fully occupied by various MIPs, while the B-tubule is relatively hollow. A series of Tektin5 proteins in different conformations reinforce the helix bundle composed by Tektin1 to Tektin4, and other MIPs with different periodicities form intricate interaction networks to anchor the Tektin bundle onto the tubulin wall of A-tubule. In B-tubule, we found the MIPs like CCDC105/TEX43/CFAP77 attaching to the ribbon and SPACA9 bound to the inner wall may play the roles to reinforce the structural stability of B-tubule. More importantly, by comparing DMT structures from the intact and deformed mouse sperm axonemes, we revealed the importance of MIPs to resist external force, where A-tubule due to its intricate interaction networks of MIPs exhibits a significant stronger structural stability than B-tubule.

Sperm undergoes a complex and delicate swinging process to harmonize the different components of its axoneme, enabling it to swim gracefully. The primary driving force behind axoneme motion is generally believed to be associated to ODA. The tail of ODA is permanently attached to the DMT A-tubule, while its microtubule-binding domain (MTBD) dynamically binds and releases the B-tubule. The affinity of dynein for the microtubule can be modulated by local geometrical alterations of the microtubule and its associated proteins on the surface^54^. Therefore, the structural differences between the A- and B-tubules might be connected to the mechanochemical cycle of dynein, requiring additional investigations. Furthermore, the A-tubule requires enhanced stability as the foundational structure to ensure the successful execution of the axoneme movements. Similarly, IDA, RS, and N-DRC are all attached to the A-tubule, and the stability of the A-tubule guarantees the coordination of each component during axoneme movement. Therefore, the stability of the A-tubule serves as an essential structural foundation for sperm to accomplish the intricate swimming process, and the numerous MIPs within the A-tubule collectively ensure this structural stability.

In both primary cilia and motile cilia, the tip region is notably thinner and typically only contains A-tubule^55^. In motile cilia and flagella, this design is advantageous for mechanical purposes as it reduces the necessary mechanical resistance during swimming. Intriguingly, within the proximity of the cilia tip, an incomplete or disassembling B-tubule can be observed in DMT^55^, similar to our DMT_W48_ structure. Hence, the DMT structure observed in the deformed sperm axoneme may represent the opposite process of DMT assembly during ciliogenesis, requiring further investigation. Moreover, microtubule turnover plays a crucial role in cilia function^56^, and the deformed DMT structure we observed may also represent an intermediate state of microtubule turnover. Furthermore, many disease-associated mutations have been identified in the sperm DMT MIPs (Supplementary Fig. S21), and comparing the natural and deformed DMT structures could provide a reference point for studying MIP-related ciliopathies^51^. Therefore, the different in situ structures of sperm DMT may enhance our understanding of mammalian sperm assembly and the roles of MIPs in the sperm axoneme. They could also shed light on future investigations into disease-associated mutations of MIPs.

Compared to the trachea cilia, sperm has an extremely long axoneme for swimming in the reproductive tract. In addition, the length of sperm flagella varies across different species, such as ~60 μm for sea urchin, ~60 μm for human, and ~120 μm for mouse^24,57^. The difference in the length of flagella may be related to fit with the specific environment during the fertilization process. With the longest axoneme, mouse sperm requires the DMT of its axoneme a hyper structural stability. Our in-cell structural study provides such molecular insight into the hyper structural stability of mouse sperm axonemal DMT and reveals rich MIPs as well as intricate interaction networks in the A-tubule lumen, which are quite different from sea urchin sperm^58^. Long-term evolutionary processes may potentially result in a correlation between the length of sperm axonemes and the abundance of MIPs within DMT. In line with this, the axoneme of sperm is longer than that of trachea cilia, and the viscosity of the reproductive tract mucus is higher than that of the respiratory tract mucus, suggesting that sperm faces greater resistance during swimming^21,59^. Therefore, it needs a more stable MIPs assembly system to protect the entire axoneme from external force and complete the fertilization process.

Compared with the recent preprint study on the isolated DMT of bovine sperm using cryo-EM SPA method, we observed conserved axonemal structure in mammalian sperm^28^. However, in bovine sperm, the SPACA9 exhibited a 48 nm periodicity with five groups of four SPACA9 proteins situated between the protofilaments B02 to B06, and one group of five SPACA9 proteins located between the protofilaments B02 to B07, forming a 5-4-4-4-4-4 pattern, which is in contrast to the 5-3-3-4-4-4 pattern of mouse sperm DMT observed in our study here, suggesting a species-specific arrangement of SPACA9, which is worthy of further investigation. Moreover, the mouse sperm DMT contains fewer Tektin5 proteins than the bovine counterpart. In mouse sperm DMT, only one Tektin5-6 protein is present in each 48 nm repeat, while two copied are found in bovine sperm (referred to as Tektin5E), warranting further studies.

Notably, a recent structural study using DMTs isolated from mouse sperm flagella has provided insights into the polymorphic Tektin5 network and multiple sperm-specific MIPs in mouse sperm^51^. The improved resolution of their DMT structure identified more MIPs compared to our own, including FAM166D, TEX37, PPP1R32, ODF3A, FAM183B, CFAP90, TEX49, CFAP68, SMRP1, and TEPP. It suggested that our model is only partially complete due to the relatively limited resolution. With the exception of RIBC2 and SPACA9, the MIPs in our models are similar with their high-resolution counterparts, thereby validating our in situ structural analysis and modeling. One of the two RIBC2 proteins in our model was further identified as RIBC1 in their high-resolution structure^51^. In particular, by maintaining the sperm sample in a more native environment, our structure reveals the complete arrangement of SPACA9 in the B-tubule. Additionally, our DMT structure of deformed sperm samples has illuminated the different roles of MIPs in maintaining the overall structures of A- and B-tubules. These findings suggest that in situ and in vitro structural analysis can complement one another, offering valuable insights into the regulation mechanism of sperm axoneme motility.

In our DMT_F96_ map, densities attributable to the IDA, ODA, N-DRC, RS1, RS2, and RS3 can be identified. Thus, cryo-FIB, cryo-ET, and STA have been shown as crucial research techniques for investigating the intricate structure of sperm axoneme in its natural environment. These methods hold great potential to advance our comprehension of the motile mechanism in mammalian sperm. The aberration of axoneme has been closely associated with asthenospermia and infertility disorders^36–38,60^. Exploring the in situ architecture of sperm axoneme would shed new light on the underlying causes of these conditions, and its integration with clinical medicine could offer valuable insights for disease management. With the further efforts including technology developments, it is foreseeable that a complete high resolution “9 + 2” architecture of the sperm axoneme will be resolved, providing in-depth understanding into molecular mechanism of sperm swimming and genetic diseases of infertility.

## Materials and methods

### Mouse sperm extraction

To extract mouse sperm, 200 μL sperm capacitive solution (Supplementary Table S6) was taken as drops in a 35 mm dish, covered with mineral oil, and placed at 37 °C in a 5% CO_2_ incubator for more than half an hour. Twelve-week-old C57 male mice were sacrificed by cervical dislocation, and the epididymis was dissected. The sperm was taken out under a stereoscope and the sperm pellets were placed in a capacitive solution. The sperm pellets were placed in the incubator for more than half an hour until the sperm pellets were completely dispersed. The sperm solution was taken out and placed in 1.5 mL Eppendorf tube and temporarily stored on ice before further experiments.

The animal experiments were performed in the Laboratory of Animal Center of Institute of Biophysics, Chinese Academy of Sciences, in accordance with the National Institutes of Health Guide for the Care and Use of Laboratory Animals and according to guidelines approved by the Institutional Animal Care and Use Committee at Institute of Biophysics with Dr. Guangxia Gao as chairman of the board.

### Sample preparation for the intact axoneme of mouse sperm

Freshly extracted sperms were centrifuged under 4 °C, 400 G (Thermo Scientific Legend Micro 17 R) for 5 min. The sediment of every 100 μL of the sperm solution was re-suspended carefully into 100 μL pre-cooled PBS on ice, then diluted 5.5 times with PBS right before use. Cryo-EM grid (Quantifoil R3.5/1, Au 200 mesh) was glow discharged using Gatan Solarus for 60 s. The sperm sample was vitrified using Leica EM GP or Leica EM GP2. 2.7 μL of PBS diluted sample was applied onto the grid, followed by immediate blotting for 2–5 s at 100% relative humidity and 4 °C, then plunge frozen into liquid ethane cooled to –186 °C, then stored in liquid nitrogen before cryo-FIB milling.

Sperm lamellae were prepared using a Aquilos 2 SEM (ThermoFisher Scientific). First, the sample was coated with a platinum layer for 10 s, followed by a coat of organometallic platinum layer for 15 s, then followed by a final coat of platinum layer for 10 s using default settings. After selecting optimum positions for milling, a stepwise tilting and milling manner was performed to obtain final lamellae: (1) tilt to 10°, set current to 300 pA, mill a 1.5 μm gap of 13 μm in width, and mill two trenches with a width of 1 μm, with a distance of 3 μm to both sides of the 13 μm gap, (2) tilt to 10.5°, set current to 100 pA, mill a gap of 800 nm from the topside and of 12.75 μm in width, (3) tilt to 9.5°, mill a gap of 500 nm from the downside and of 12.75 μm in width, (4) tilt to 10°, set current to 50 pA, mill a gap of 300 nm from both side and of 12.25 μm in width, (5) set current to 30 pA, and with extensive tilting to 10.2° or 9.8° to reach a final gap of 80 nm from both side with 12 μm in width. All tilt angle aforementioned are defined as the relative tilting angle with respect to the grid. Once the optimum thickness had been reached, the grid was sputtered with a final layer of platinum at 30 KV, 10 mA for 1–3 s, then stored in liquid nitrogen before data collection in TEM.

### Sample preparation for the deformed axoneme of mouse sperm

Freshly extracted sperms were centrifuged under 4 °C, 400 G (Thermo Scientific Legend Micro 17 R) for 5 min. The sediment of every 200 μL of the sperm solution was re-suspended carefully into 100 μL pre-cooled PBS on ice, then diluted 20 to 40 times with PBS before further experiments. Cryo-EM grid (Quantifoil R1.2/1.3, Au 200 mesh) was glow discharged using Gatan Solarus for 60 s right before use. The diluted sperm solution was mixed at a ratio of 1:1 with 10× concentrated 10 nm protein A-coated gold fiducials (Electron Microscopy Sciences). 2.7 μL of the mixture was applied onto the grids and blotted for 1.5 to 2.5 s in 100% relative humidity and 4 °C, then plunged into liquid ethane for vitrification, and transferred into liquid nitrogen before data collection, this process was performed by using Vitrobot Mark IV (ThermoFisher Scientific).

### Cryo-ET tilt series acquisition

Both cryo-FIB milled and directly frozen grids were mounted onto Autoloader in Titan Krios G3 (Thermofisher Scientific) 300 KV TEM, equipped with a Gatan K2 direct electron detector (DED) and a BioQuantum energy filter. Tilt series were collected under a magnification of ×81,000, resulted in a physical pixel size of 1.76 Å, or 0.88 Å under super-resolution mode, in K2 DED. Before data collection, the pre-tilt of the sample was determined visually, and the pre-tilt was set to be 10° or –9° to match the pre-determined geometry caused by loading grids. The total dose was set to 3.5 electrons per square angstrom per tilt, fractioned to 10 frames in a 1.2 s exposure, and the tilt range were set to be between –66° to +51° for –9° pre-tilt or –50 to +67° for +10° pre-tilt, with a 3° step, resulting in 40 tilts and 140 electrons per tilt series. The slit width was set to be 20 eV, with the refinement of zero-loss peak after collection of each tilt series, and nominal defocus was set to be –1.8 to –2.5 μm. For directly frozen sperm samples, the tilt range was set to be –60° to +60°, starting at 0°, with all other parameters unchanged. All tilt series used in this study were collected using a dose-symmetry strategy-based beam-image-shift facilitated acquisition scheme, by in-house developed scripts within SerialEM software^61–63^.

### Image processing of F-dataset

After data collection, all fractioned movies were imported into Warp for essential processing including motion correction, Fourier binning by a factor of 2 of the super-resolution frames, CTF estimation, masking platinum islands or other high-contrast features, and tilt series generation^64^. Subsequently, the tilt series underwent automatic alignment using AreTOMO^64,65^. The aligned tilt series were visually examined in IMOD, and any frames of low quality (such as those obstructed by the stage or grid bar, containing apparent crystalline ice, or displaying an apparent jump) were removed to create new sets of tilt series in Warp. The new sets of tilt series underwent a second round of AreTOMO alignment. Then frames of low quality were again removed using the same criteria as in the first round. The new tilt series then underwent a third round of automatic alignment, continuing this process until no frames required removal. After tilt series alignment, those tilt series with less than 30 frames or completely failed in alignment were kept from further processing^65,66^. The alignment parameters of all remaining tilt series were transferred back to Warp, and initial tomogram reconstruction was done at a pixel size of 14.08 Å in Warp.

After examining all the data, we found that about one in seven (108/689) of the FIB-milled tomograms contained intact “9 + 2” axonemes. Then, DMT particles was manually picked using the filament picking tool in Dynamo, by picking the start and end points of each DMT filament and separating each crop point by 8 nm along the filament axis^67^. The 3D coordinates and two of three Euler angles (except for in-plane rotation) were generated automatically by Dynamo, then transferred back to Warp for exporting of sub-tomograms^64,67,68^.

Sub-tomogram refinement was done in RELION 3.0 or 3.1, transform of RELION’s star file and Dynamo’s table file was done by ABTT package, mask generation was done by combination of Dynamo and/or RELION^69,70^. First, all particles were reconstructed in box size of 64^3^ voxels with pixel size of 14.08 Å, and an initial reference was generated by low-pass filtering at 60 Å of directly average of all picked-out particles. Then a *K* = 1 3D classification with restrictions of the first two Euler angles (*--sigma_tilt 3* and *--sigma_psi 5* in RELION) was done for 100 iterations. Then these aligned parameters were transferred back to Warp to export sub-tomograms in box size of 72^3^ voxels with pixel size of 7.04 Å. To find the 16 nm repeat particles, a classification job using A-tubule MIP mask was employed. By referring to the obvious Tektin5-4 density within the A-tubule lumen, the particles corresponding to the 16 nm repeat were chosen, while any duplicated particles were removed using Dynamo. Then one round of 3D auto refinement was done in RELION, with a mask covering solely the A-tubule of 16 nm repeat of DMT.

From this point on, the data processing was split into different branches of A/B tubule of 16 nm/48 nm repeats. For 16 nm repeat maps, the aligned parameter was transferred back to M for multi-particle refinement, and the resolution reaches the Nyquist limit. Then the sub-tomograms corresponding to A- and B-tubule were reconstructed in box size of 128^3^ voxels with 3.52 Å pixel size by M, and extensive refine and 3D classification were performed in RELION^71^. When resolution of these particles stopped increasing, the aligned parameters were transferred back to M to export A- and B-tubule sub-tomograms in box size of 216^3^ voxels with 1.76 Å pixel size, then extensive auto refinement in RELION and M refinement was done, which reached a final resolution of 4.5 Å for A-tubule MIP and 6.5 Å for B-tubule.

For 48 nm repeat maps, a mask inside A-tubule lumen of 16 nm repeat map was applied to find the map with 48 nm periodicity, then sub-tomograms in box size of 200^3^ voxels with 3.52 Å pixel size were exported for auto refinement in RELION. Then, six different sets of masks were applied evenly onto A- and B-tubule, and auto refinement in RELION and M refinement was done using these masks, reaching final resolutions of 6.5 to 7.5 Å in box size of 256^3^ voxels with 1.76 Å pixel size. 96 nm repeat maps were generated by classifying 48 nm repeat map with a local mask covering RS and N-DRC regions (Supplementary Figs. S2–S5).

To estimated axoneme circularities in F-dataset and W-dataset, the axonemes that contains 9 manually picked DMT were taken for calculation. This resulted in 108 axonemes for the F-dataset and 96 axonemes for the W-dataset. Subsequently, the DMT-center distance was calculated by measuring the distance between the axoneme center and each DMT. The axoneme circularity was estimated by determining the ratio of the smallest DMT-center distance to the largest DMT-center distance.

### Image processing of W-dataset

The data processing of W-dataset is basically the same as that of F-dataset. The 16 nm repeat map was generated by auto refinement at binned 2 level in RELION and M refinement to reach a final resolution of 7.9 Å. The 48 nm repeat map was auto-refined in RELION and further refined in M with a final resolution of 8.6 Å (Supplementary Figs. S6 and S7). We failed to acquire a 96 nm repeat maps in W-dataset. The data processing statistics were summarized in Supplementary Table S1.

Mask creation at different image processing stages was done by a combination of UCSF Chimera, Dynamo, and RELION, and converting of Dynamo table file or RELION star file or tilt series alignment files was done by a wrap of automatic tools of sub-tomogram data processing^67–70,72,73^.

Visualization of cryo-EM maps, image making, and rendering of refined models was performed using either UCSF Chimera, UCSF ChimeraX, or 3D Protein Imaging^48,73,74^. For difference map calculation, DiffMap in CCP-EM software package were used^75^.

### MS

Mouse sperm for MS identification was cleaned with PBS and then centrifuged at 2000× *g* for 5 min. We then treated the sperm with five different buffers (B1: 0.1% Triton; B2: 0.6 M NaCl; B3: 8 M Urea; B4: 10% SDS; B5: 0.1% Triton+8 M Urea). For 10% SDS, we added the buffer to sperm precipitation and heated it at 95 °C for 5 min. The other buffers were incubated at room temperature for 10 min after sperm precipitation. The treated sperm was centrifuged at 2000× *g* for 10 min, and 20 µl supernatant was taken for SDS-PAGE. Coomassie brilliant blue was stained and decolorized using eStain L1 protein stain instrument (L00657C) (Supplementary Fig. S8).

After decolorization, the strips were enzymolized with trypsin overnight, and then the peptides were extracted with acetonitrile of different concentrations in multiple steps. The peptide mixture obtained by enzymolysis was analyzed by liquid chromatography-tandem MS. Then SEQUEST HT search engine of Thermo Proteome Discoverer (2.4.1.15) was used to search and identify proteins in the Uniprot_proteome_mouse (update-07/2022) protein library database. The search results were authenticated by MS on nanoLC-Orbitrap Exploris 480.

### Model building

Modeling was based on each asymmetric unit of tubulin dimer and MIPs. Since there were no available structures of mouse DMT proteins in the PDB database when this manuscript was prepared, the AlphaFold^76^ predicted models were always used as the starting model. Two different strategies were used to generate the final model in our study.

On one hand, several proteins have homologs previously identified in bovine trachea DMT^15^. To enhance the reliability of modeling, the predicted structure of each corresponding mouse protein was substituted with a poly-alanine model. Subsequently, it was fitted into the density and manually adjusted using Coot^77^. Following this, the findMySequence program^30^ was utilized to search for the protein candidate within the mouse protein database (17,141 reviewed sequences in UniProtKB). For proteins that the findMySequence program confirmed identification with the highest confidence score, their predicted AlphaFold structures were flexibly fitted into the density using ISOLDE plugin within ChimeraX^48^. The models were manually adjusted using COOT and further refined through pheix.real_space_refine in Phenix^78^. This process was iterated several times to generate the final model. The models of tubulin α/β, CFAP20, CFAP276, CFAP52, EFCAB6, EFHC1, EFHC2, PACRG, Tektin1 to Tektin4, and TEKTIP1 were all built in this manner due to their higher local resolutions and map qualities (Supplementary Fig. S11, Tables S3 and S4). For α-tubulin family proteins, which have several outputs in the findMySequence program, we chose the highest-ranked protein candidate for modeling based on the MS results list (Supplementary Dataset S1). For proteins where the findMySequence program failed to identify a meaningful candidate, the homologous proteins in bovine trachea DMT were directly assigned to the structure. Their AlphaFold structures were flexibly fitted into the density using ISOLDE plugin within ChimeraX, and were manually adjusted using COOT. Subsequently, we refined them further using pheix.real_space_refine in Phenix. This process was iterated several times to generate the final model. The models of NME7, CFAP161, MNS1, CFAP53, RIBC2, CFAP141, CFAP95, CFAP107, SPAG8, Pierce1, Pierce2, CFAP21, FAM166A, ENKUR, CFAP126, CFAP45, and CFAP210 were all built using this approach due to their lower local resolutions.

On the other hand, for those proteins not involved in the bovine trachea DMT structure, we selected the top 1500 candidates from the MS results list (Supplementary Dataset S1). We manually fitted their AlphaFold-predicted models into the local density to identify the best match. In cases where multiple candidates matched the density, we selected the highest-ranked protein based on the MS results list. Subsequently, we fine-tuned the model using COOT and refined it with pheix.real_space_refine in Phenix. This process was iterated several times to generate the final model. The models of CCDC105, DUSP21, Tektin5, SPACA9 were all built using this approach. For example, the models of all Tektin isotypes were found to fit well within the Tektin5 densities, but Tektin5 exhibited the highest-ranked score (26th) on the MS results list (Supplementary Table S2), surpassing the other Tektin isotypes (Tektin1 to Tektin4, ranked 82nd to 185th). As a result, we assigned these densities to be Tektin5. Notably, the assignments of CCDC105, DUSP21, Tektin5-1 and Tektin5-2 were confidently confirmed by the findMySequence program, owing to their significantly higher local resolutions and map qualities. However, some local densities within the structure were too small, and the reliability of the manual fitting results was limited. To address this, we refer to the preprint report on the composition of bovine sperm axoneme^28^, and manually fitted the AlphaFold predicted structure into the local density. The models of FAM166C, TEX43, and CFAP77 were built in this manner.

### Supplementary information

Supplementary information

Supplementary Video 1

Supplementary Video 2

Supplementary Video 3

Supplementary Dataset 1

## Data Availability

The sub-tomogram averaged cryo-EM maps for A tubule in 16 nm repeat, B tubule in 16 nm, A tubule (part1, 2, 3) in 48 nm repeat, B tubule (part1, 2, 3) in 48 nm repeat, section I of DMT in 96 nm repeat, section II of DMT in 96 nm repeat as well as the composite maps DMT_F16_, DMT_F48_, and DMT_F96_ in F-dataset have been deposited in the Electron Microscopy Database (EMDB) under the accession codes EMD-35210, EMD-35211, EMD-35222, EMD-35224, EMD-35225, EMD-35226, EMD-35227, EMD-35228, EMD-35231, EMD-35232, EMD-35229, EMD-35230, and EMD-35236, respectively. The sub-tomogram averaged cryo-EM maps for DMT_W16_ and DMT_W48_ have been deposited in EMDB under the accession codes EMD-35237 and EMD-35238, respectively. The atomic models of DMT in 16 nm repeat and in 48 nm repeat have been deposited in Protein Data Bank with the accession codes of 8I7O and 8I7R, respectively.
