# Supplementary material for: In-cell structural insight into the stability of sperm microtubule doublet
Source: Cell Discov. 2023 Nov 21;9:116. doi: 10.1038/s41421-023-00606-3 (PMC10663601; doi:10.1038/s41421-023-00606-3)
Supplement: Supplementary file 1 — Supplementary information [file 41421_2023_606_MOESM1_ESM.pdf]

Supplementary information

for

In-cell structural insight into the stability of sperm microtubule doublet

Linhua Tai[1, 5], Guoliang Yin[1, 2, 5], Xiaojun Huang[3], Fei Sun[1, 2, 3, 4, *] and Yun Zhu[1, *]

[1] National Key Laboratory of Biomacromolecules, CAS Center for Excellence in Biomacromolecules, Institute of Biophysics, Chinese Academy of Sciences, Beijing 100101, China.

[2] University of Chinese Academy of Sciences, Beijing 100049, China.

[3] Center for Biological Imaging, Institute of Biophysics, Chinese Academy of Sciences, Beijing 100101, China.

[4] Bioland Laboratory (Guangzhou Regenerative Medicine and Health Guangdong Laboratory), Guangzhou, Guangdong 510005, China.

[5] These authors contributed equally to this work.

1. **Supplementary Figures S1-21**
2. **Supplementary Videos S1-3**
3. **Supplementary Tables S1-6**
4. **Supplementary Dataset S1**
5. **References**

Supplementary Figures

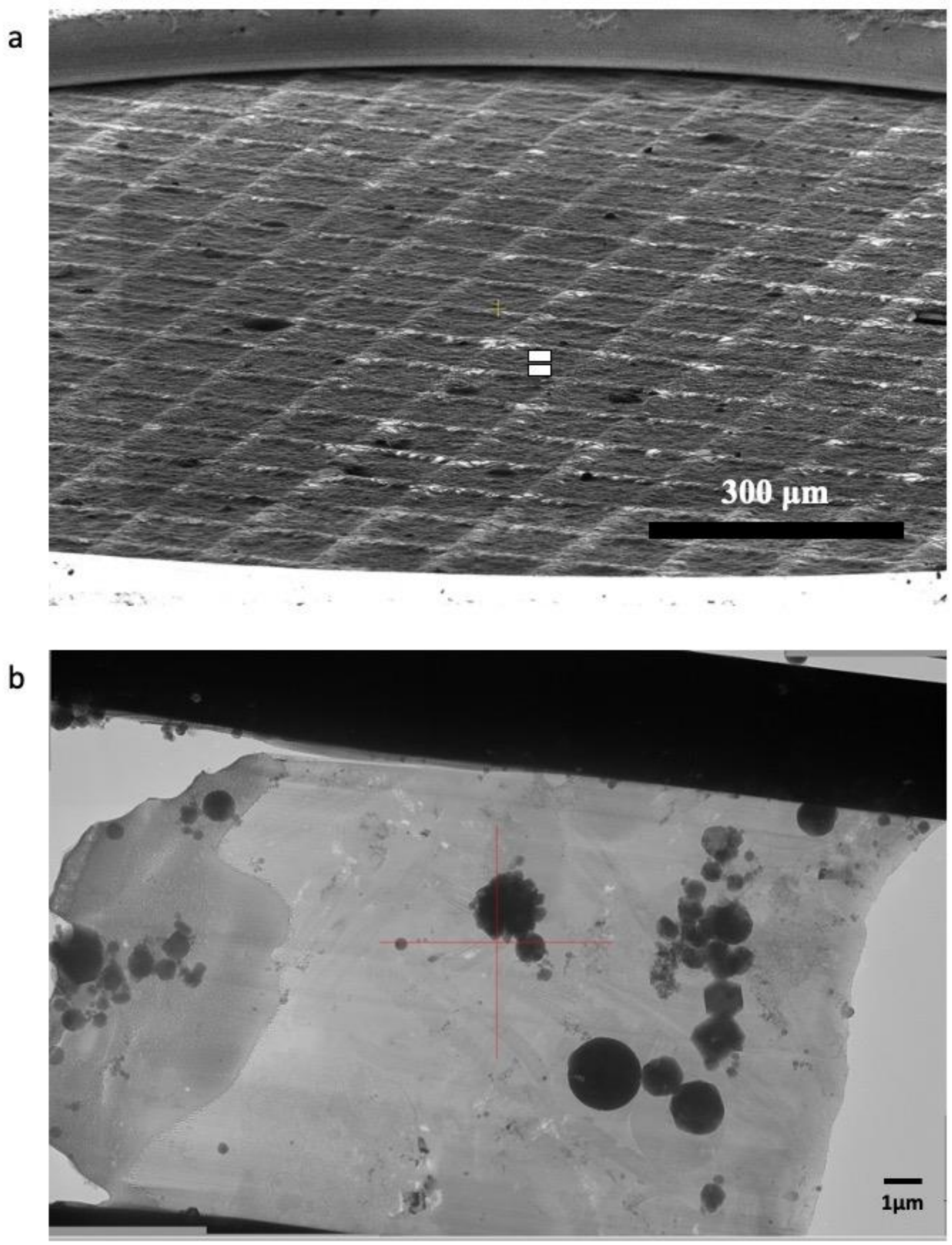

Supplementary Figure S1. Cryo-FIB milling of mouse sperm axoneme. (a) Inspection of frozen sperm on grid before FIB milling. Representative mouse sperm tails were marked with dotted lines. **(b)** A representative lamella with multiple sperm axoneme used for data collection.

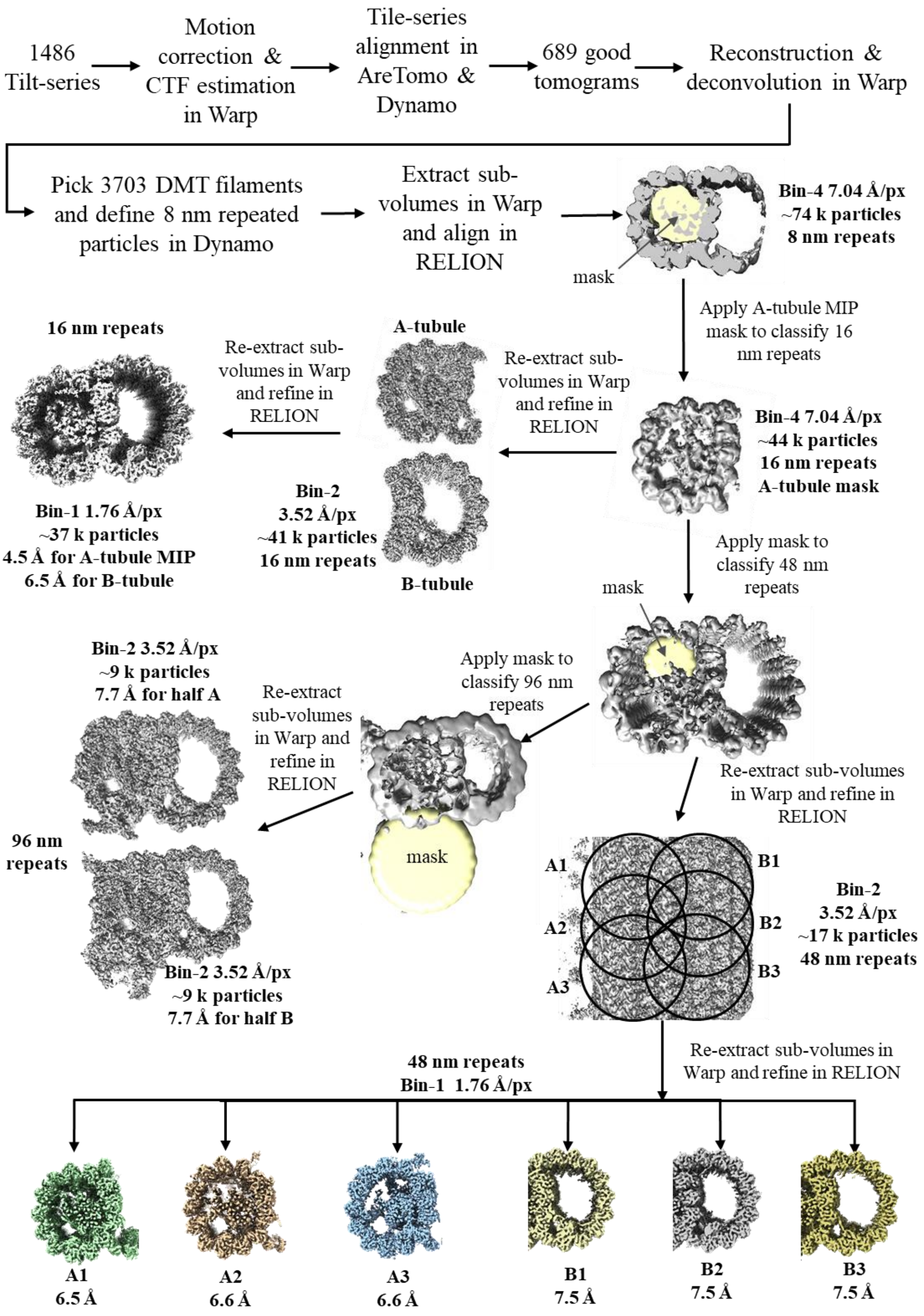

Supplementary Figure S2. Data processing procedure for F-dataset. The pixel sizes at different binning levels are indicated in angstroms per pixel (Å/px for short).

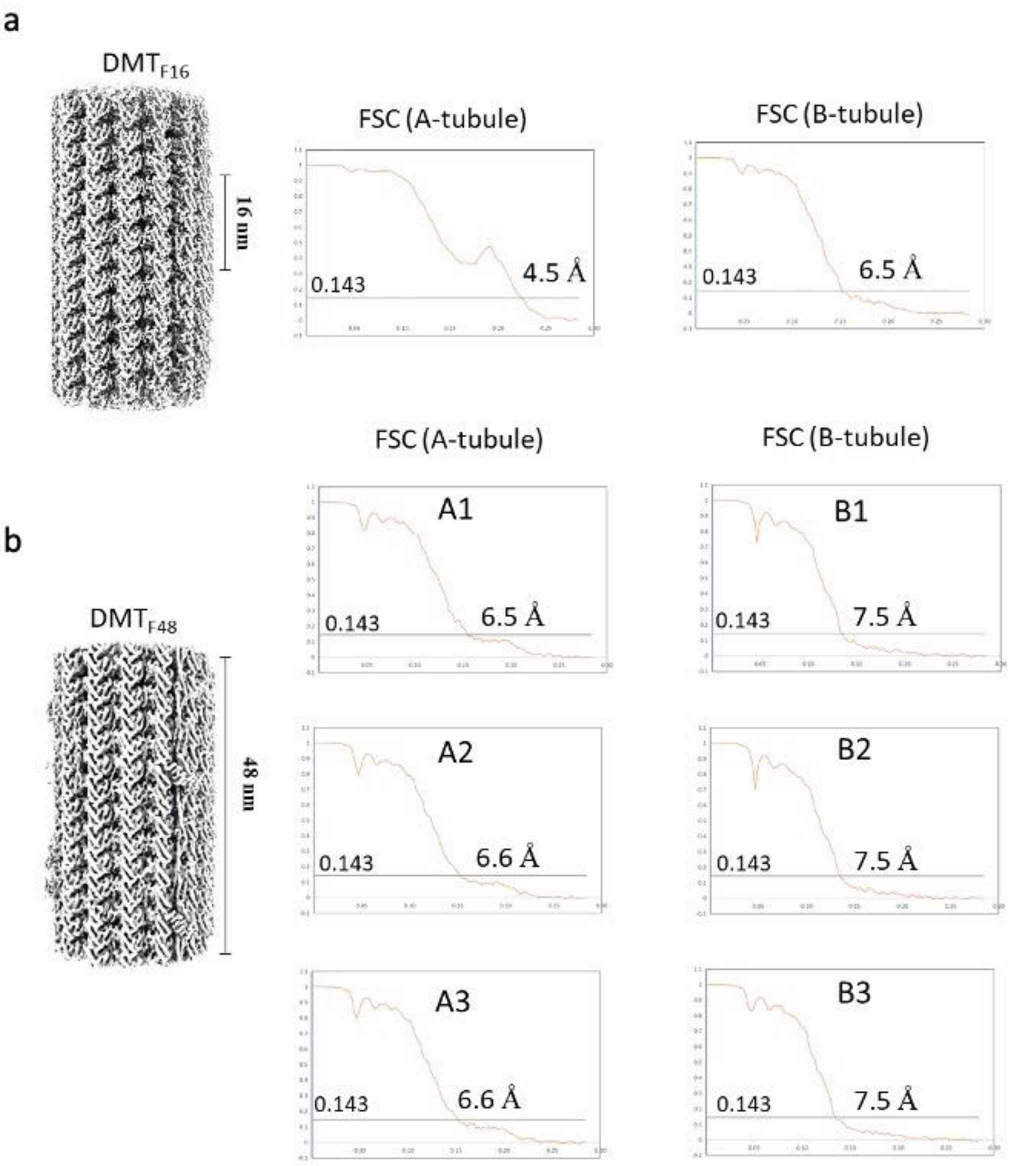

Supplementary Figure S3. Golden standard Fourier shell correlation (FSC) curves of cryo-EM maps DMT$_{F16}$ and DMT$_{F48}$ in F-dataset. (a) Cryo-EM map and FSC curves of DMT$_{F16}$. **(b)** Cryo-EM map and FSC curves of DMT$_{F48}$. The resolutions at the FSC cut-off criteria of 0.143 are indicated accordingly. The local regions, A1, A2, A3, B1, B2 and B3, are described in Supplementary Fig. S2.

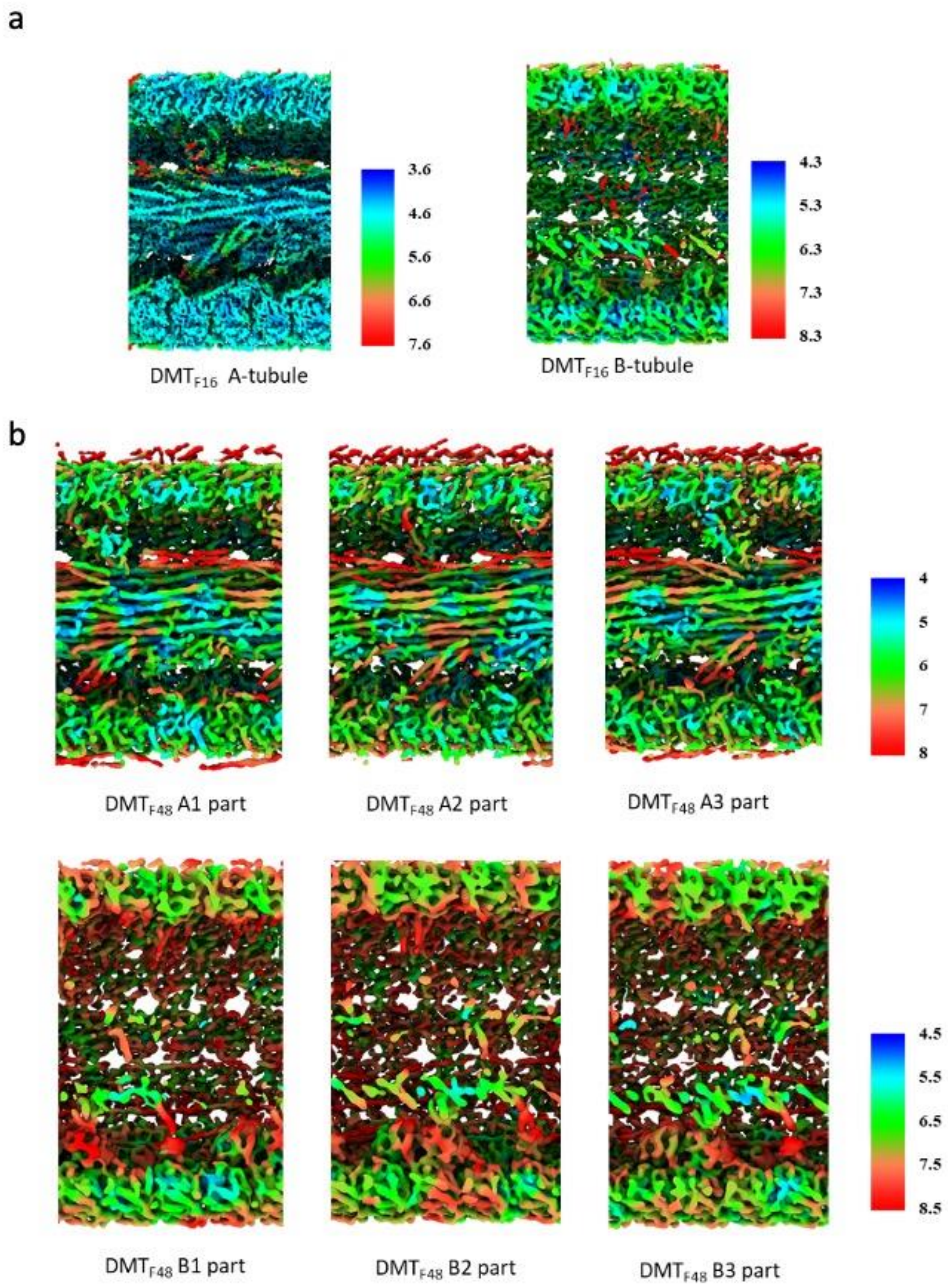

Supplementary Figure S4. Local resolution of cryo-EM maps DMT$_{F16}$ and DMT$_{F48}$ in F-dataset. (a) Local resolution of DMT$_{F16}$. **(b)** Local resolution of DMT$_{F48}$. The local regions, A1, A2, A3, B1, B2 and B3, are described in Supplementary Fig. S2.

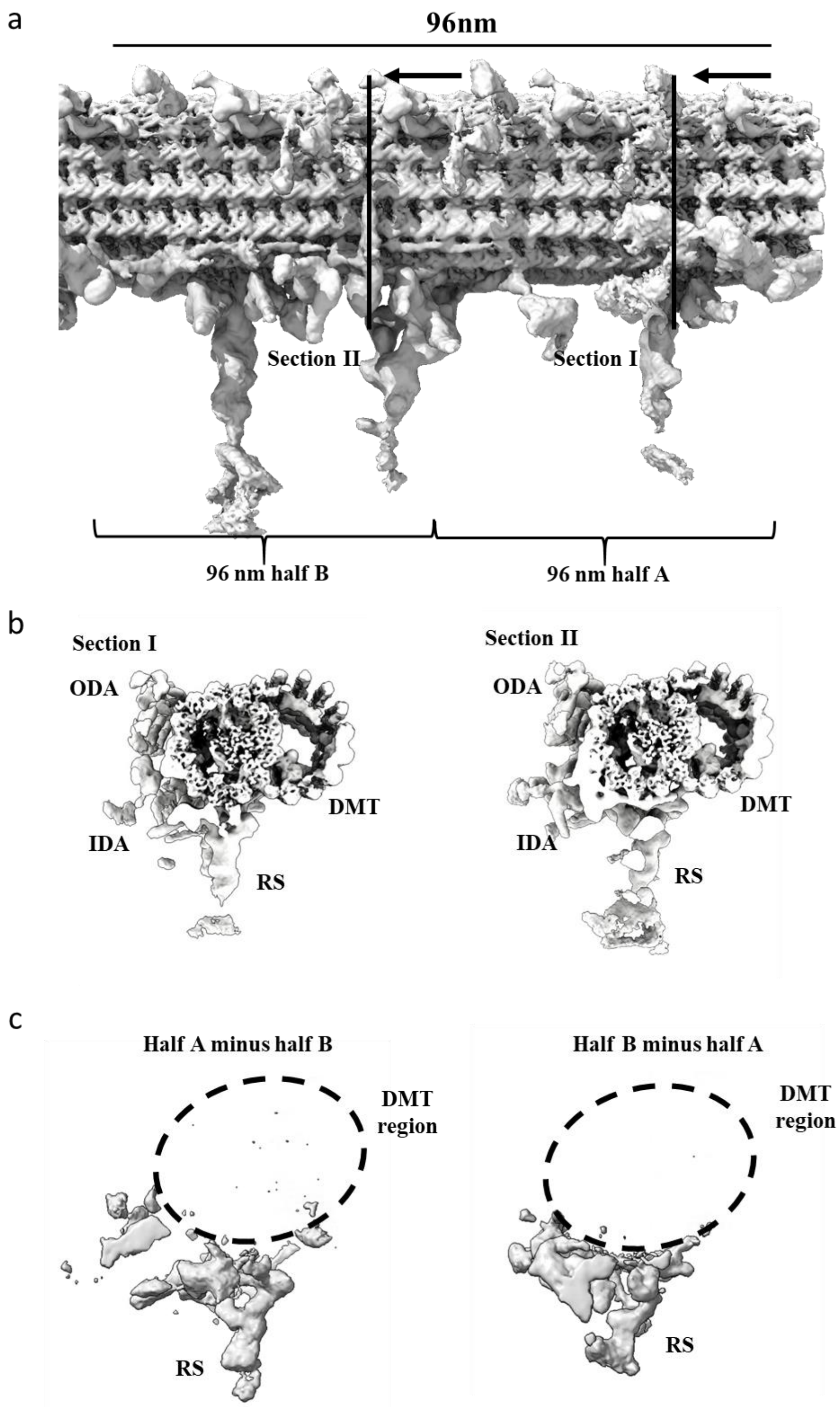

Supplementary Figure S5. Cryo-EM map of mouse sperm DMT with 96 nm repeats in F-dataset. (a) Cryo-EM map of DMT_{F96} with the side view. **(b)** Transverse sections of DMT_{F96} maps at section I and II in (a), showing the identical density of MIPs. **(c)** Difference map of two 48 nm repeats within the 96 nm map.

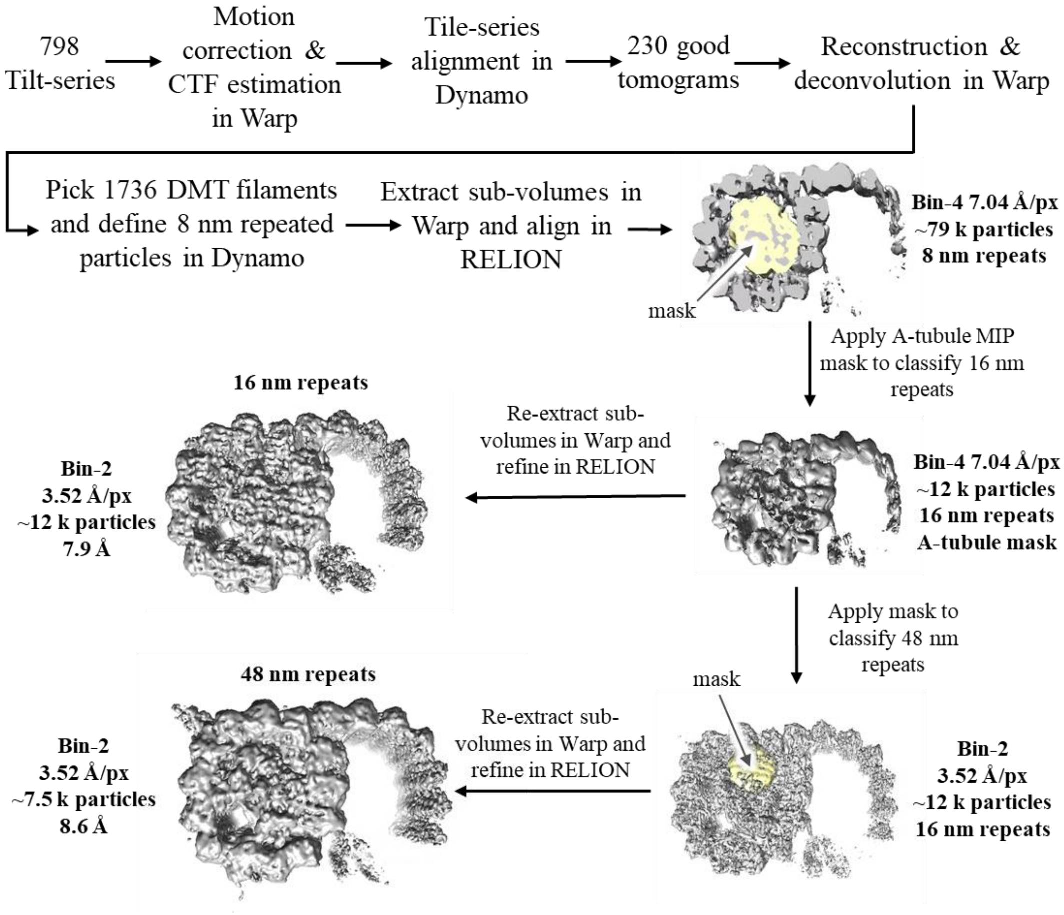

Supplementary Figure S6. Data processing procedure for W-dataset. The pixel sizes at different binning levels are indicated in angstroms per pixel (Å/px for short).

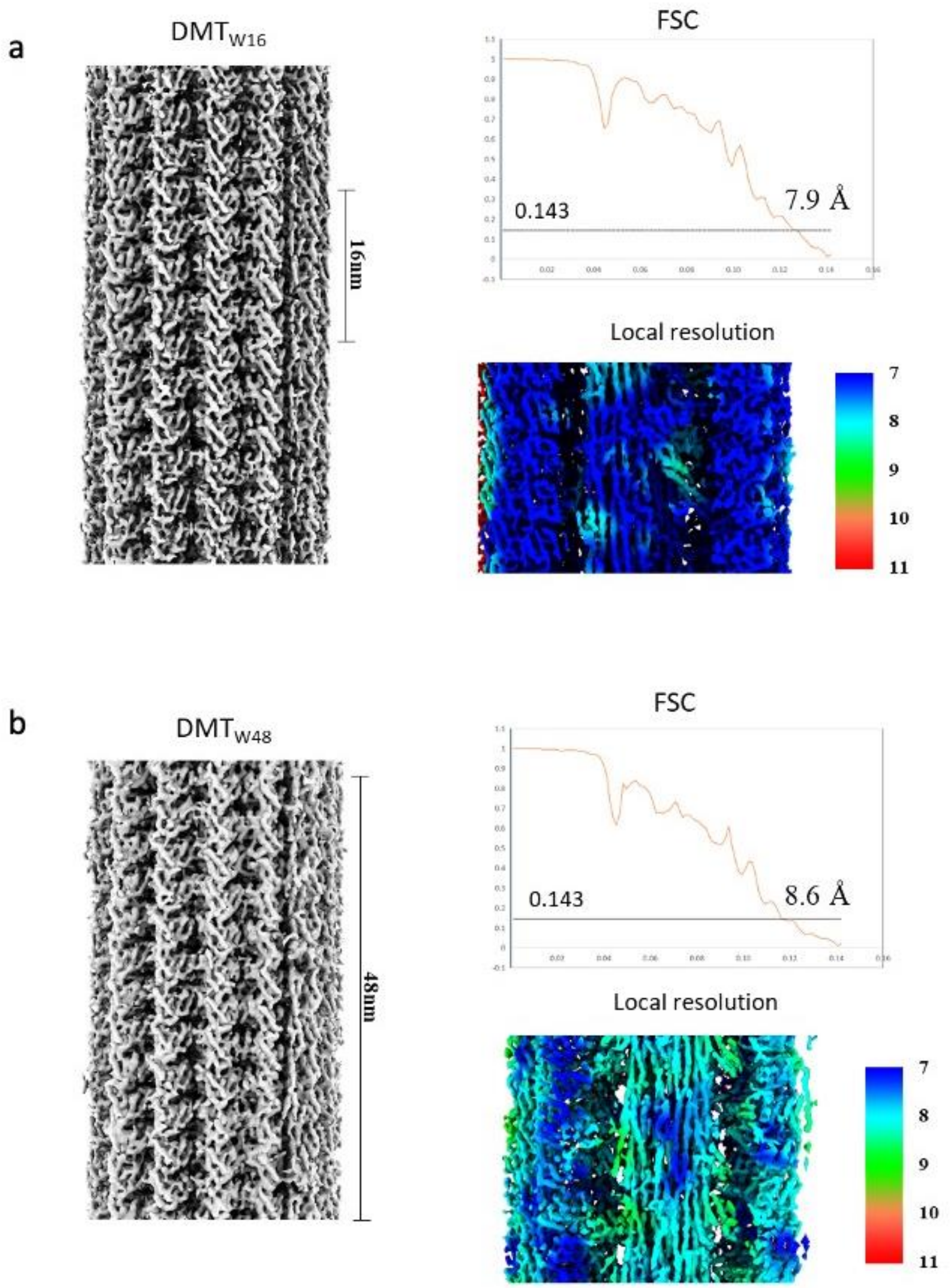

Supplementary Figure S7. Cryo-EM maps, golden standard FSC curves and local resolution analyzes in W-dataset for DMT_{W16} (a) and DMT_{W48} (b). The resolutions at the FSC cut-off criteria of 0.143 are indicated accordingly.

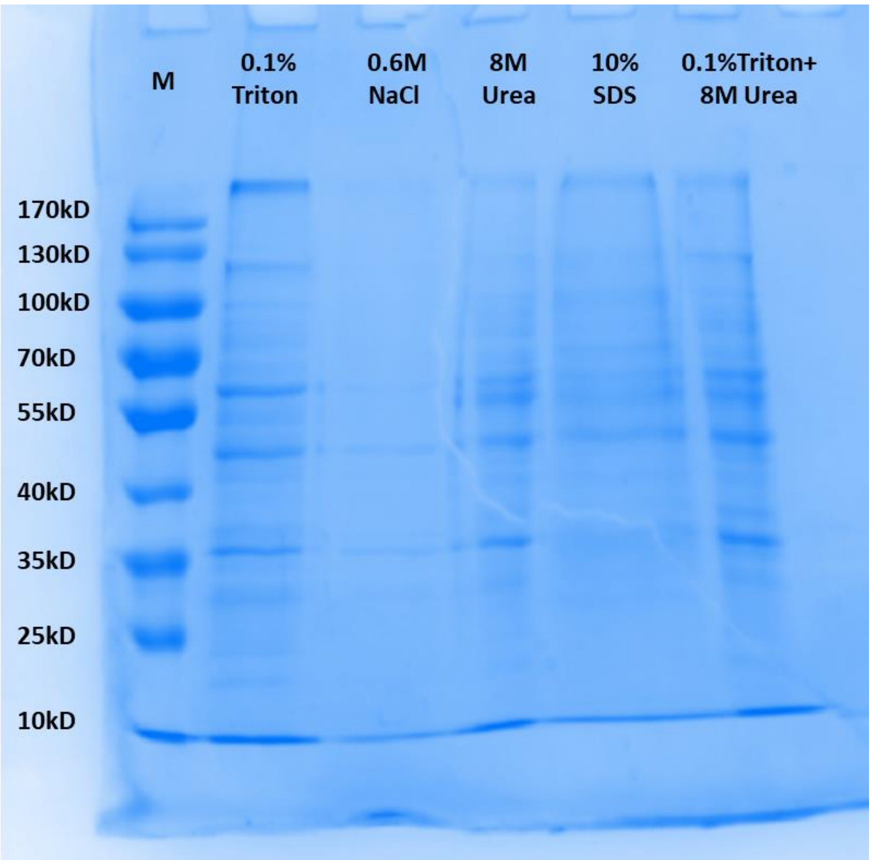

Supplementary Figure S8. SDS-PAGE of mouse sperm proteome. Mouse sperm samples were treated with different solutions and analyzed by SDS-PAGE. These SDS-PAGE bands were analyzed by mass spectrometry. Molecular weights markers (M) are indicated.

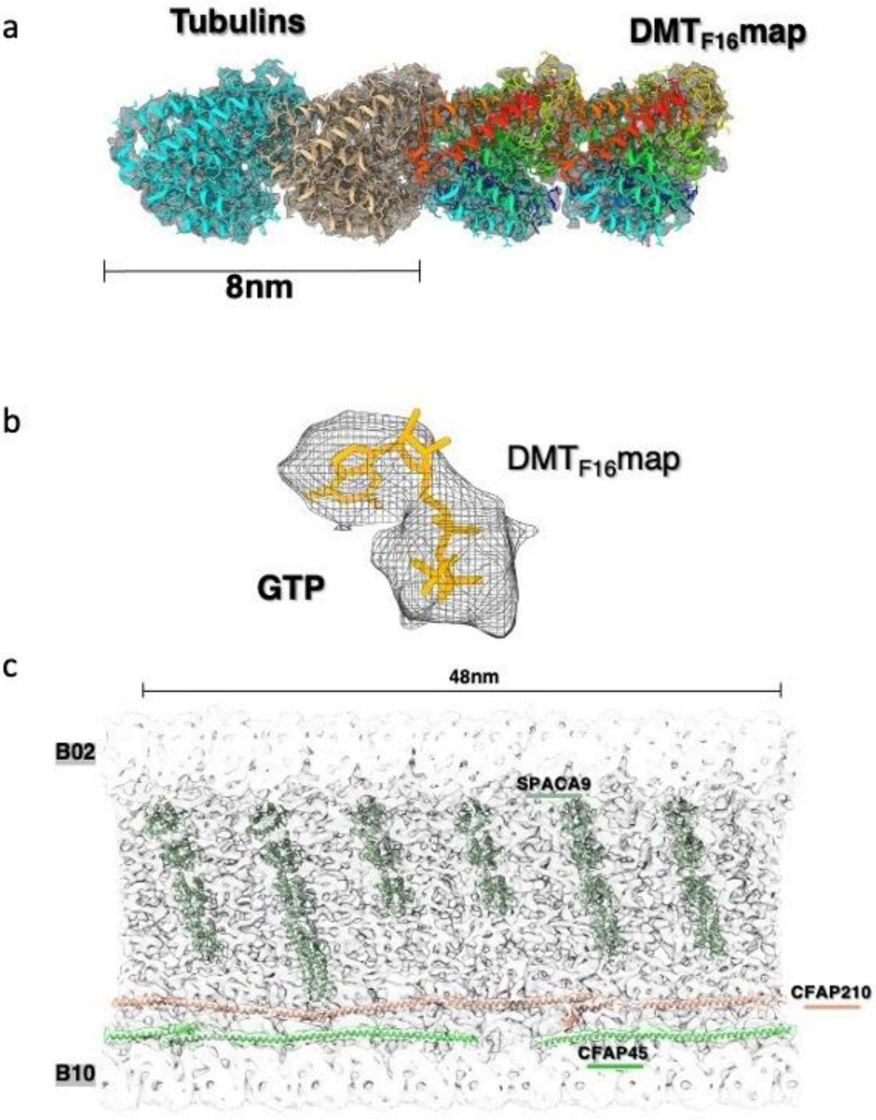

Supplementary Figure S9. The map-model fitting quality of tubulins in DMT$_{F16}$ map and the model fitting of CFAP201, CFAP45 and SPACA9 in DMT$_{F48}$ map. (a) The alpha- (colored in cyan and rainbow) and beta- (colored in wheat and rainbow) tubulins are modelled well into the DMT$_{F16}$ map. **(b)** Density map of GTP in tubulin. **(c)** CFAP201, CFAP45 and SPACA9 attach to the inner surface of B-tubule with the periodicity of 48 nm at the location between B02 and B10.

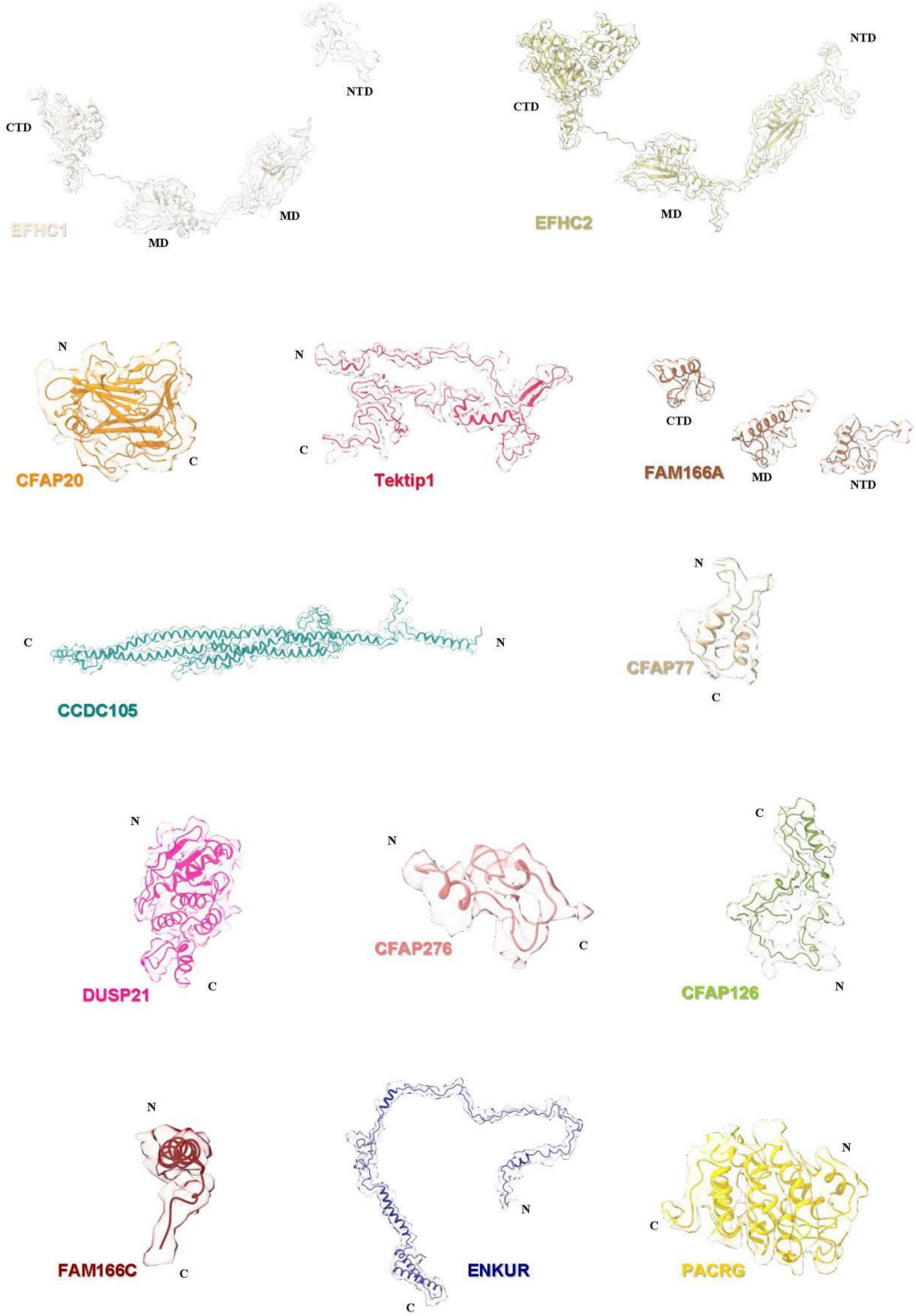

NTD
CTD
EFHC1
MD
MD
NTD
CTD
EFHC2
MD
N
C
CFAP20
N
C
Tektip1
CTD
MD
NTD
FAM166A
C
N
CCDC105
N
CFAP77
C
N
C
DUSP21
N
C
CFAP276
C
N
CFAP126
N
C
FAM166C
N
C
ENKUR
N
C
PACRG

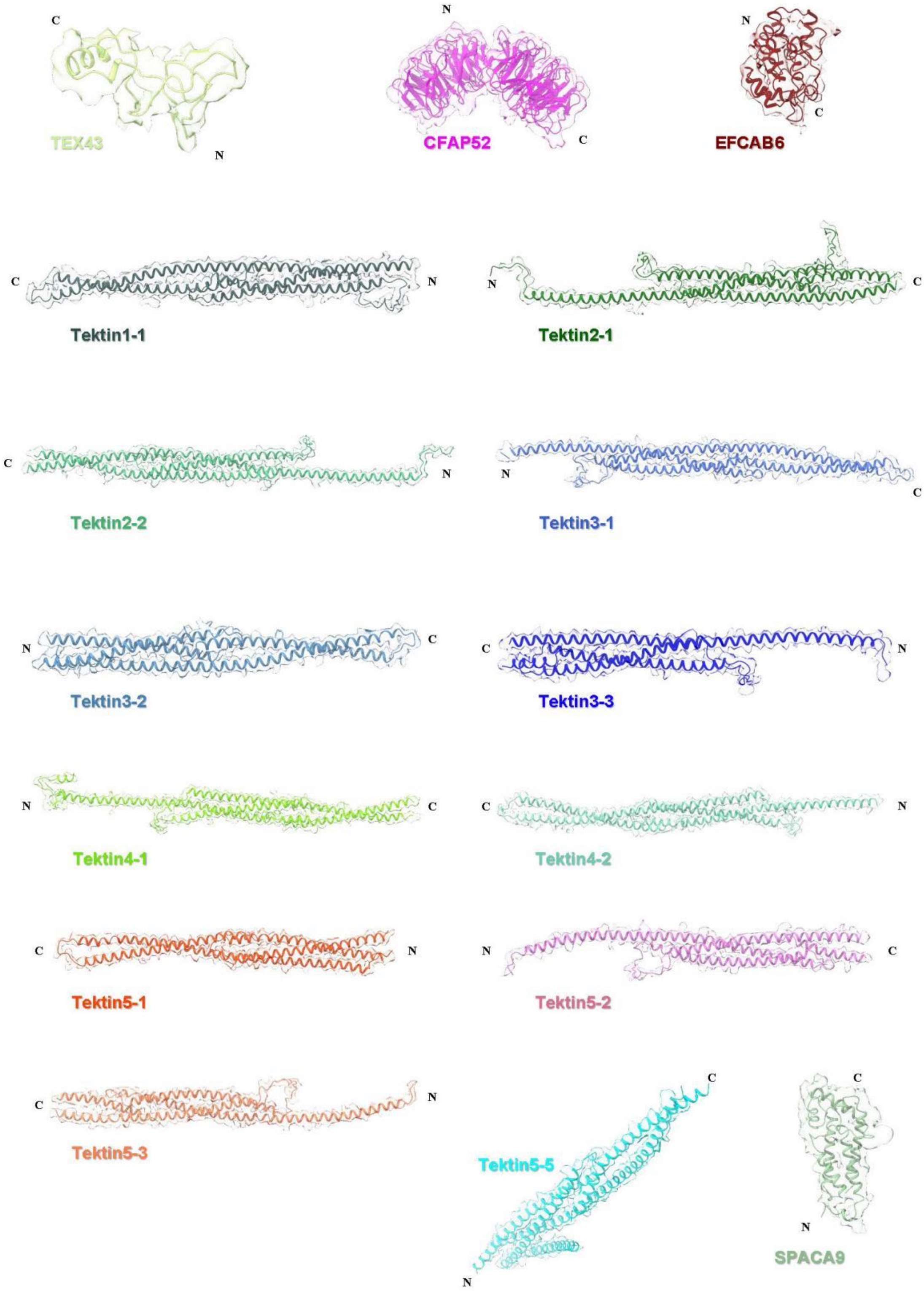

Supplementary Figure S10. Overall map-model fitting quality of MIPs in DMT$_{F16}$ map.

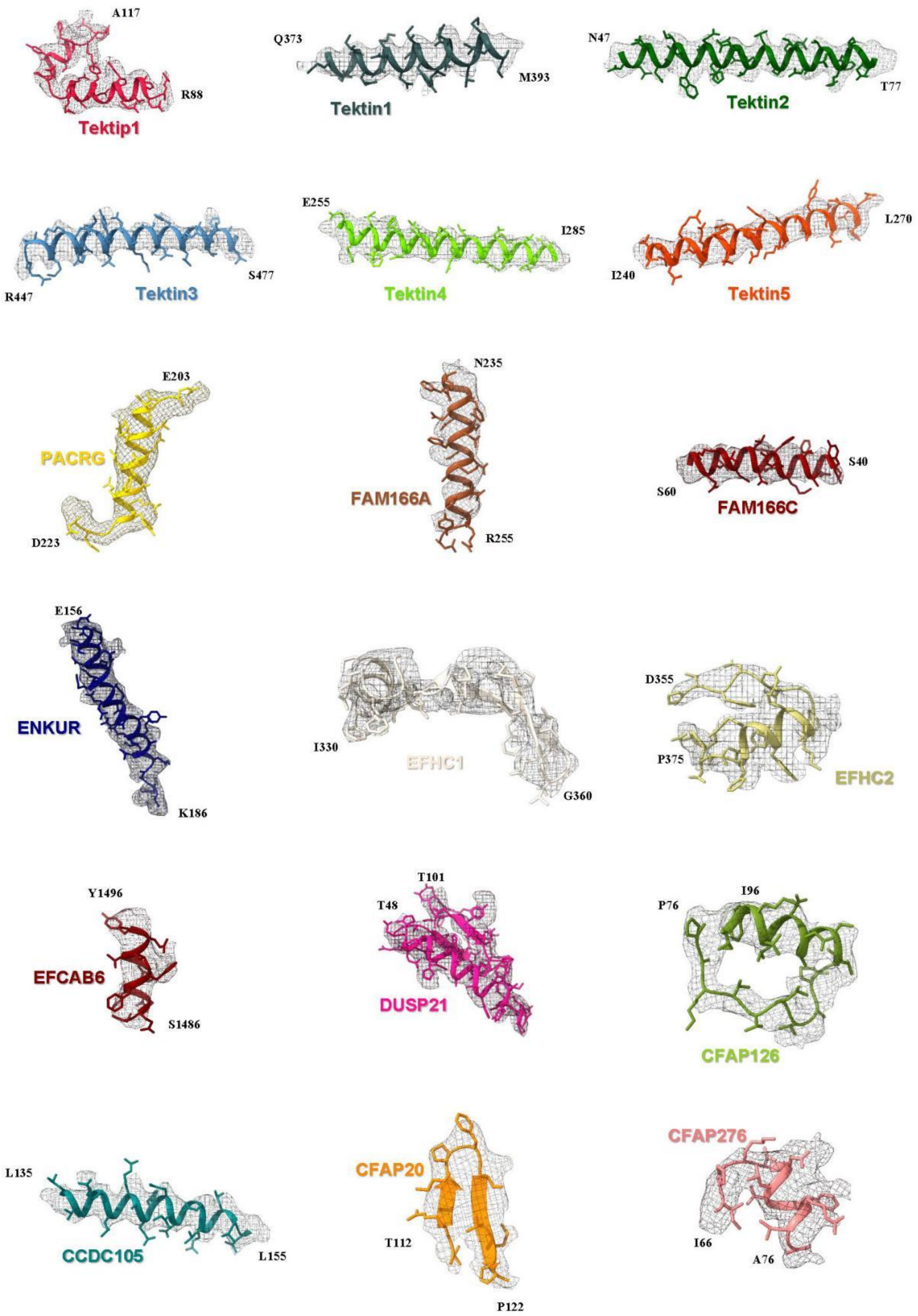

Supplementary Figure S11. Model-map fitting quality in representative local areas of MIPs in DMT_{F16} map.

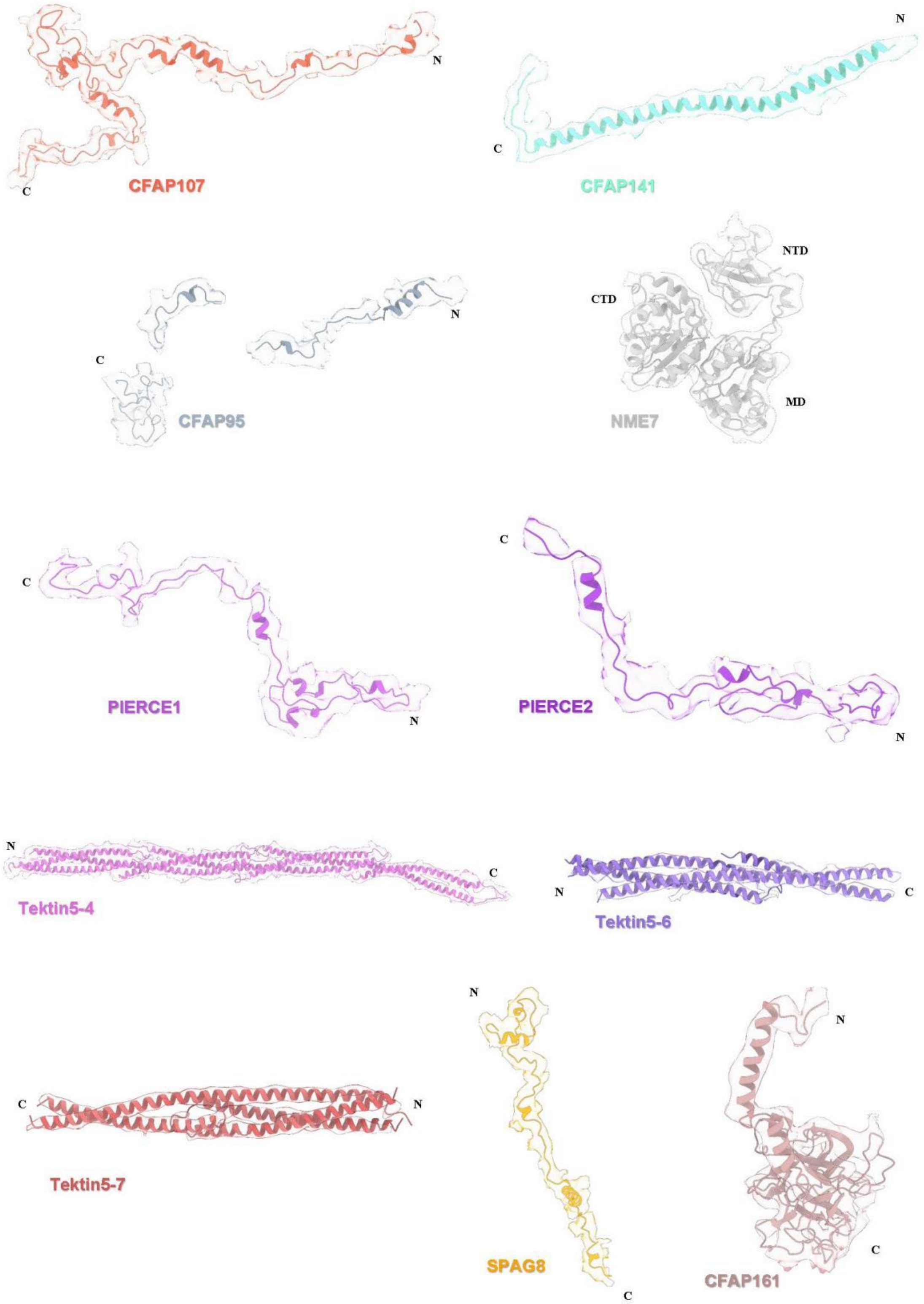
N
C
CFAP107
N
C
CFAP141
N
C
CFAP95
NTD
CTD
MD
NME7
C
N
PIERCE1
C
N
PIERCE2
N
C
Tektin5-4
N
C
Tektin5-6
C
N
Tektin5-7
N
C
SPAG8
N
C
CFAP161

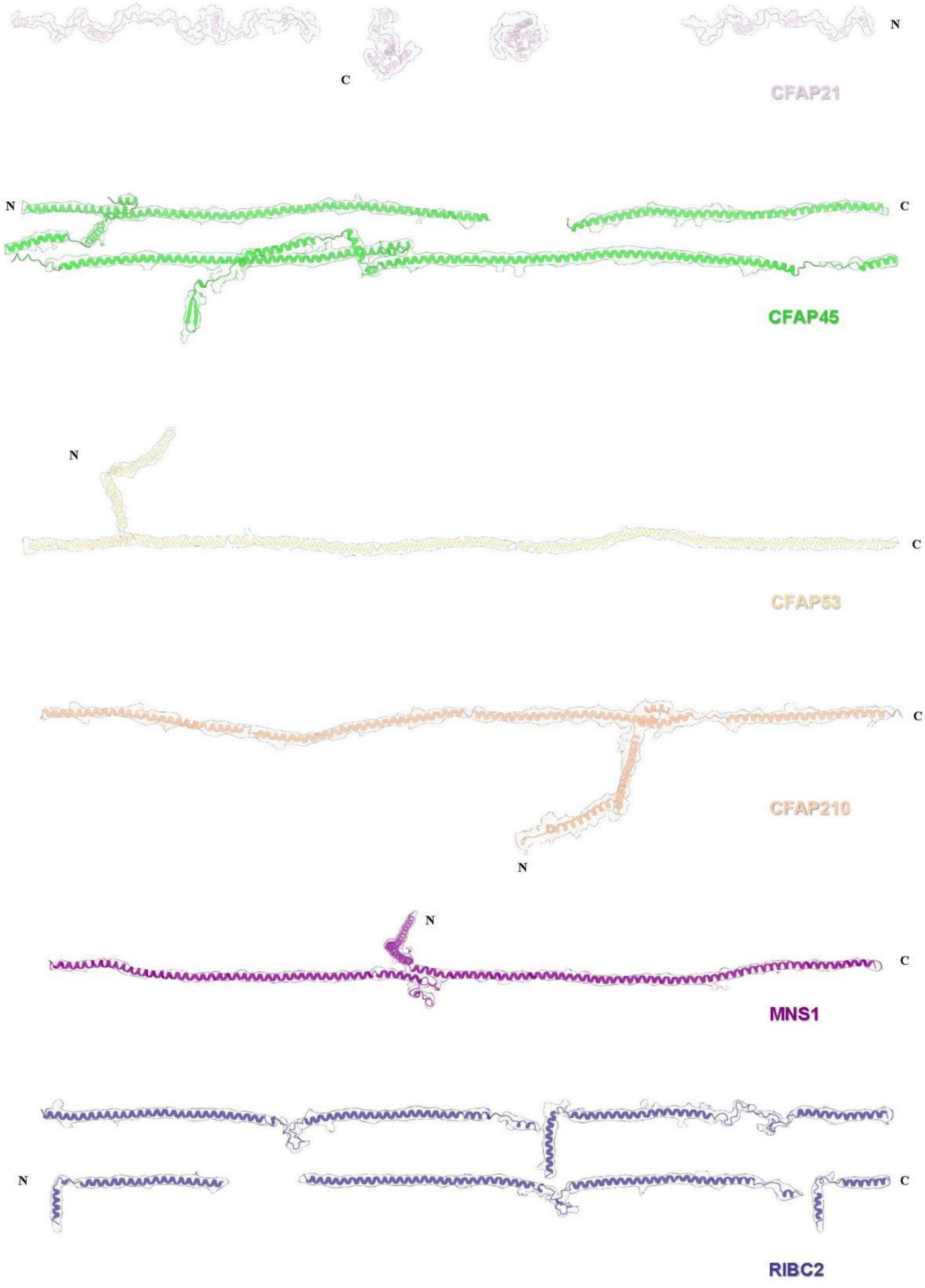

Supplementary Figure S12. Overall map-model fitting quality of MIPs in DMT$_{F48}$ map.

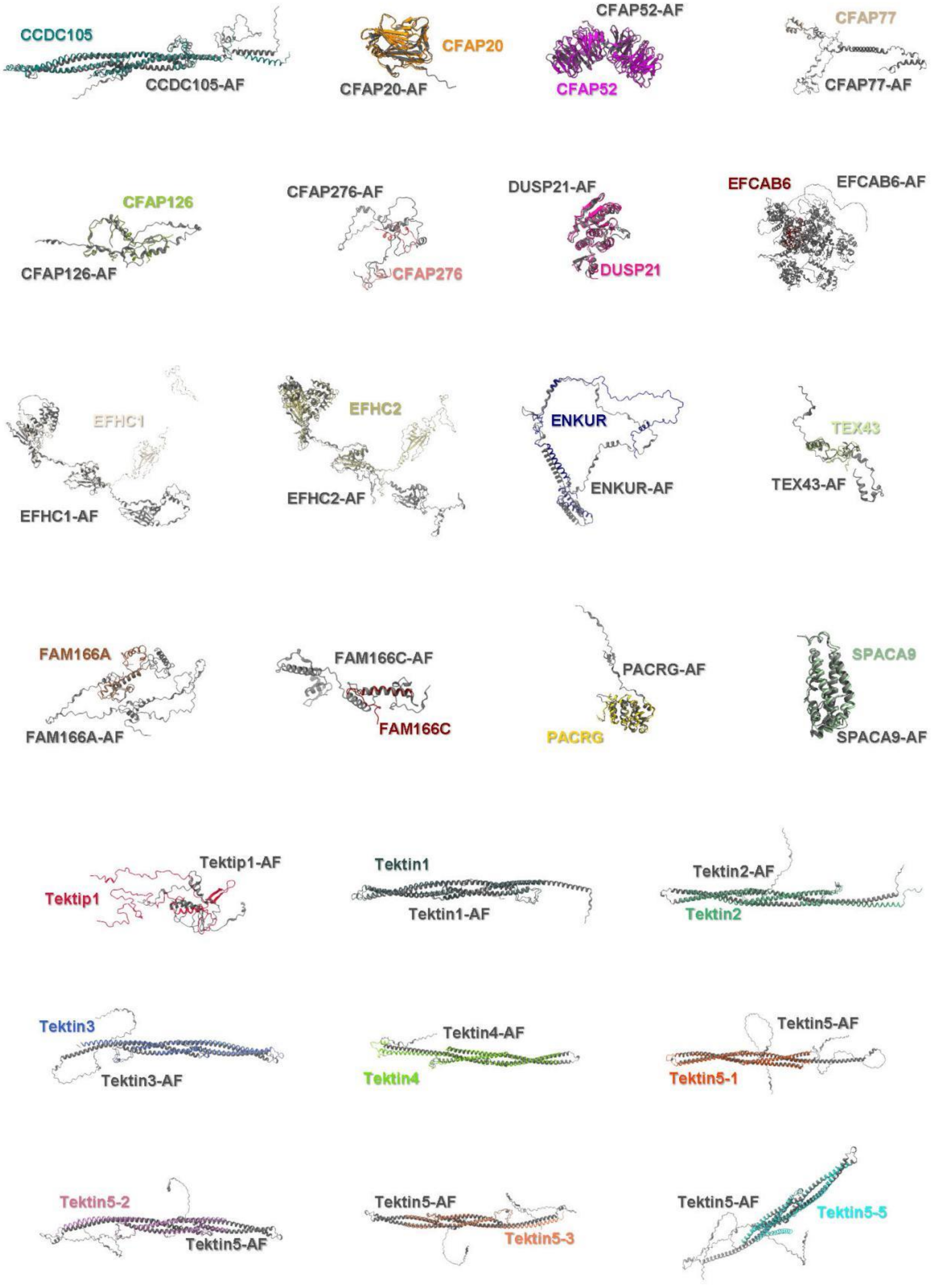

Supplementary Figure S13. Structural superpositions between AlphaFold2 predicted and final refined models of MIPs in DMT$_{F16}$ map. The model predicted by AlphaFold2 is colored in grey and designated with the suffix "-AF".

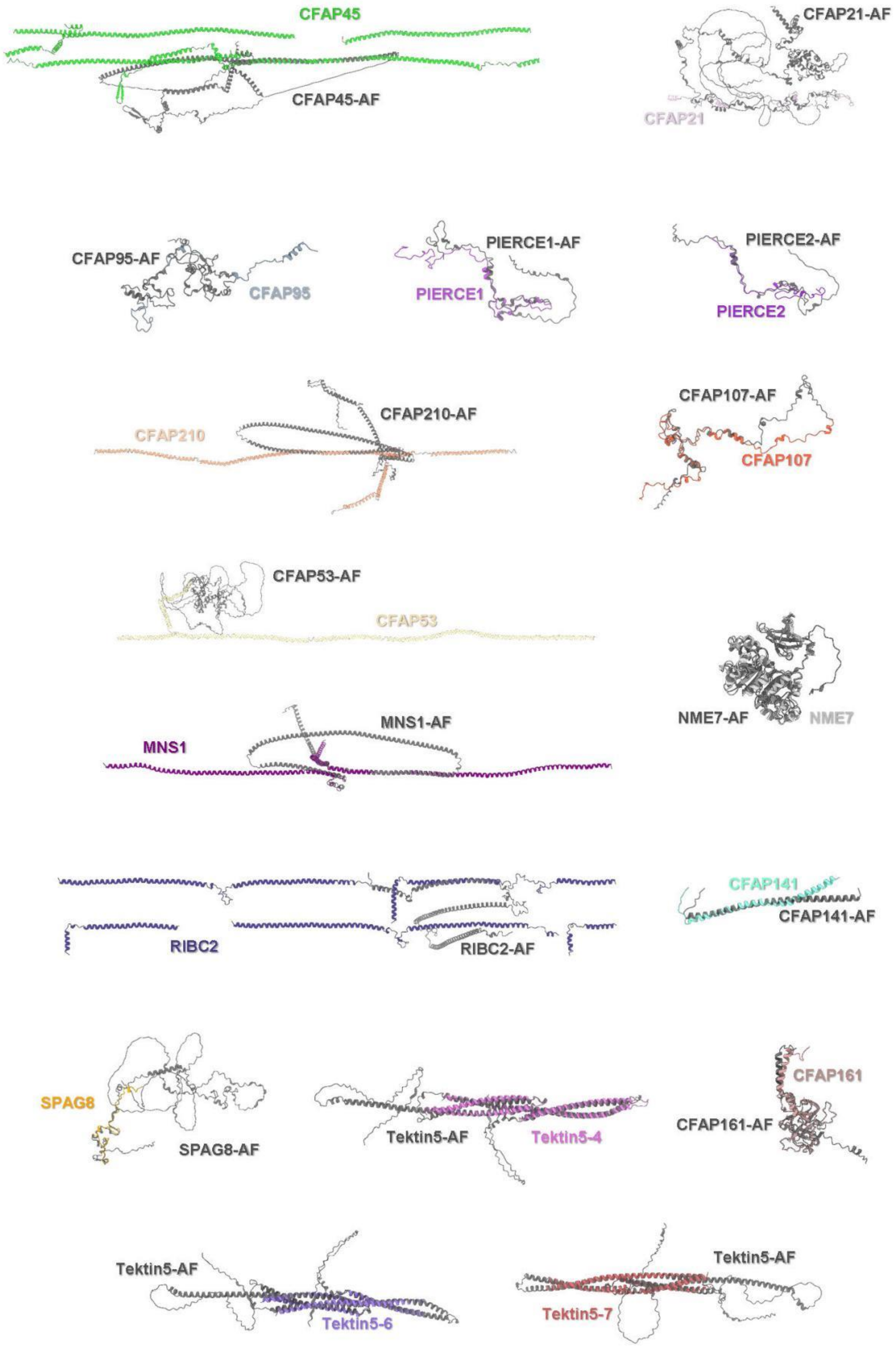

Supplementary Figure S14. Structural superpositions between AlphaFold2 predicted and final refined models of MIPs with the periodicity of 48 nm in $\mathbf{DMT_{F48}}$ map. The model predicted by AlphaFold2 is colored in grey and designated with the suffix "-AF".

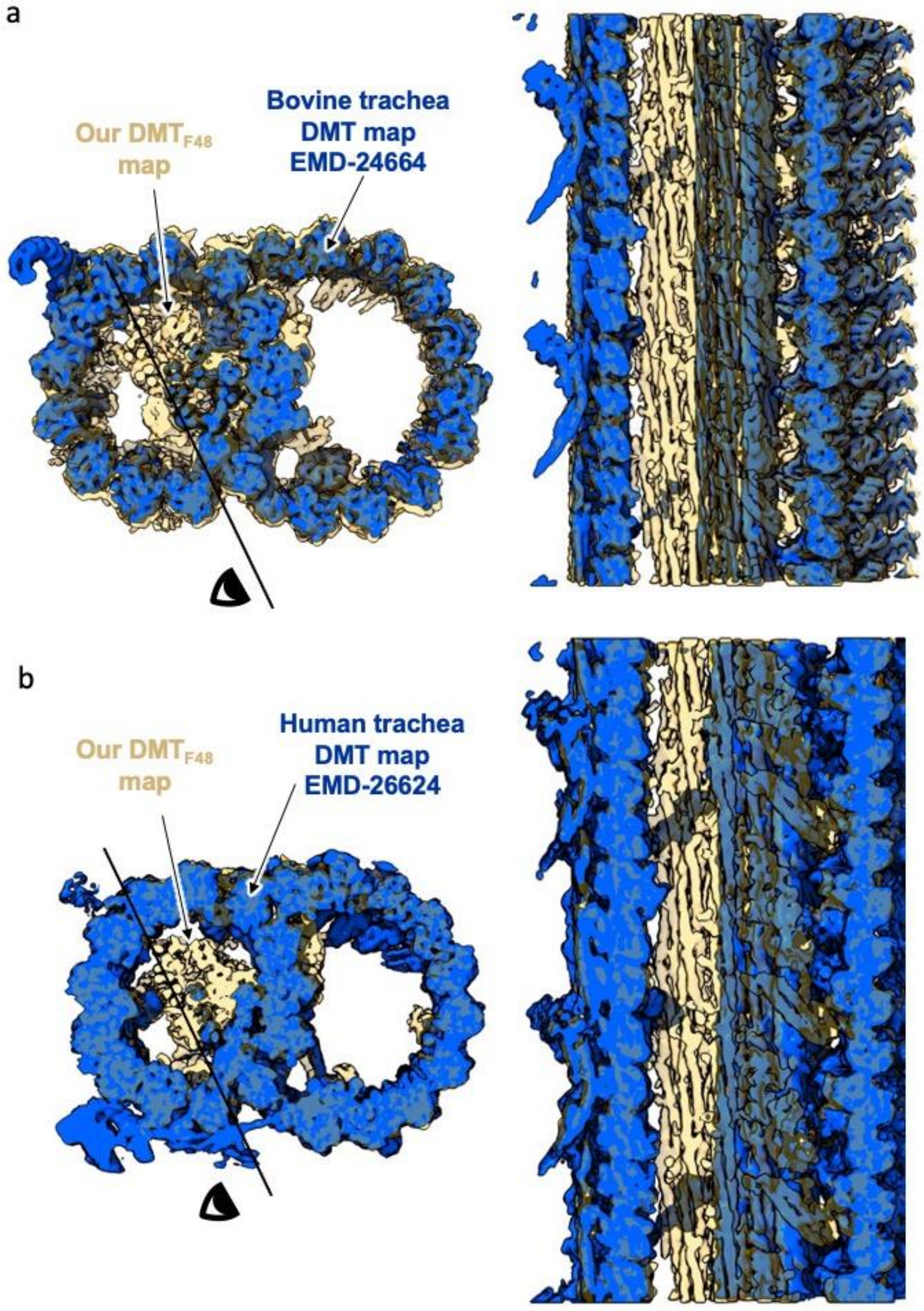

Supplementary Figure S15. Comparison of our DMT$_{F48}$ map with trachea DMT map from bovine (a) or human (b). Our DMT$_{F48}$ map (yellow) is fitted into the trachea DMT map (blue) from bovine (EMD-24664) or human (EMD-26624), and shown in transverse section and side view.

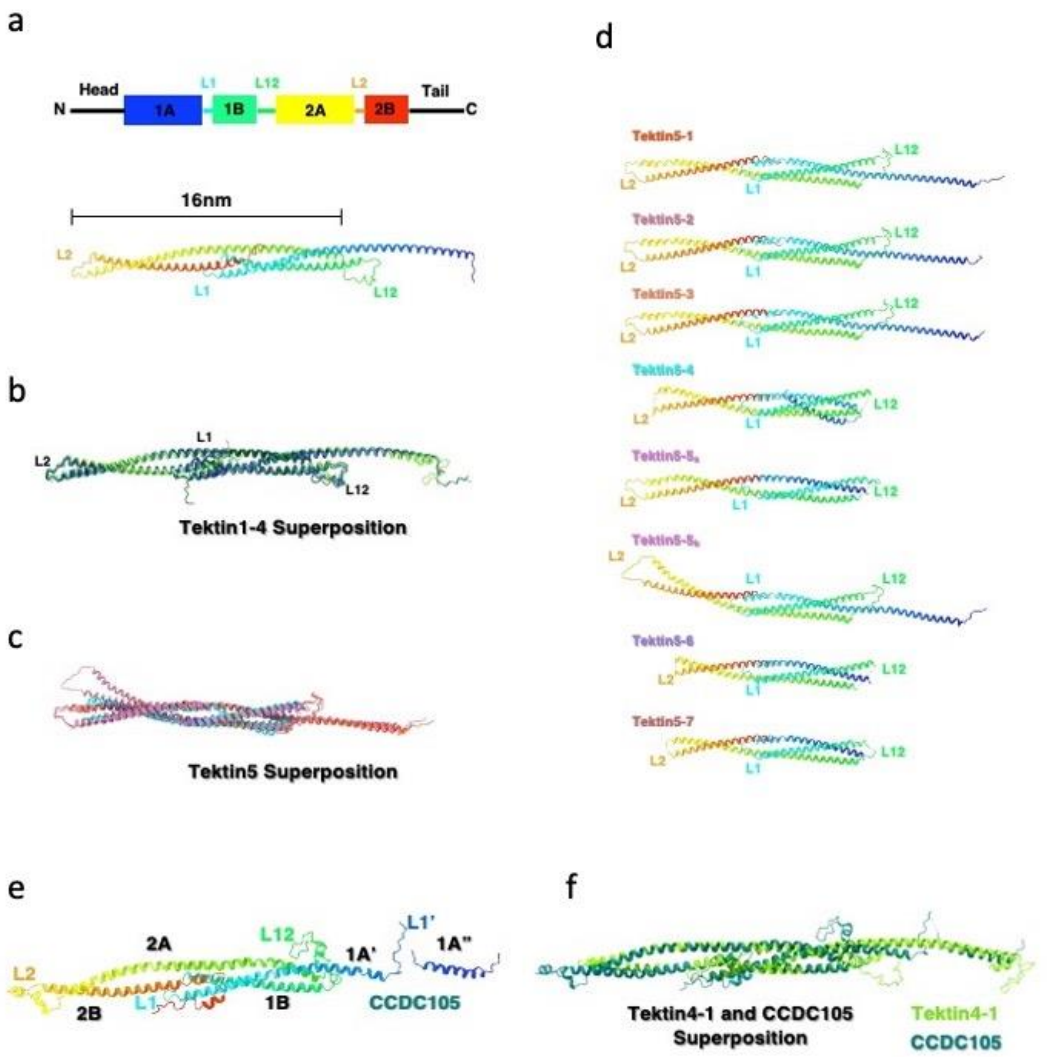

Supplementary Figure S16. Structural comparison among Tektin family proteins and CCDC105. (a) Schematic diagram of the domain distribution of Tektin family protein. **(b)** Structural superposition of Tektin1/2/3/4 proteins. **(c)** Structural comparison of Tektin5 proteins in 7 different positions (two conformations in position 5, 5a and 5b). **(d)** Structural superposition of Tektin5 proteins. **(e)** Structure of CCDC105 with domains labeled. **(f)** Structural superposition between Tektin4-1 and CCDC105.

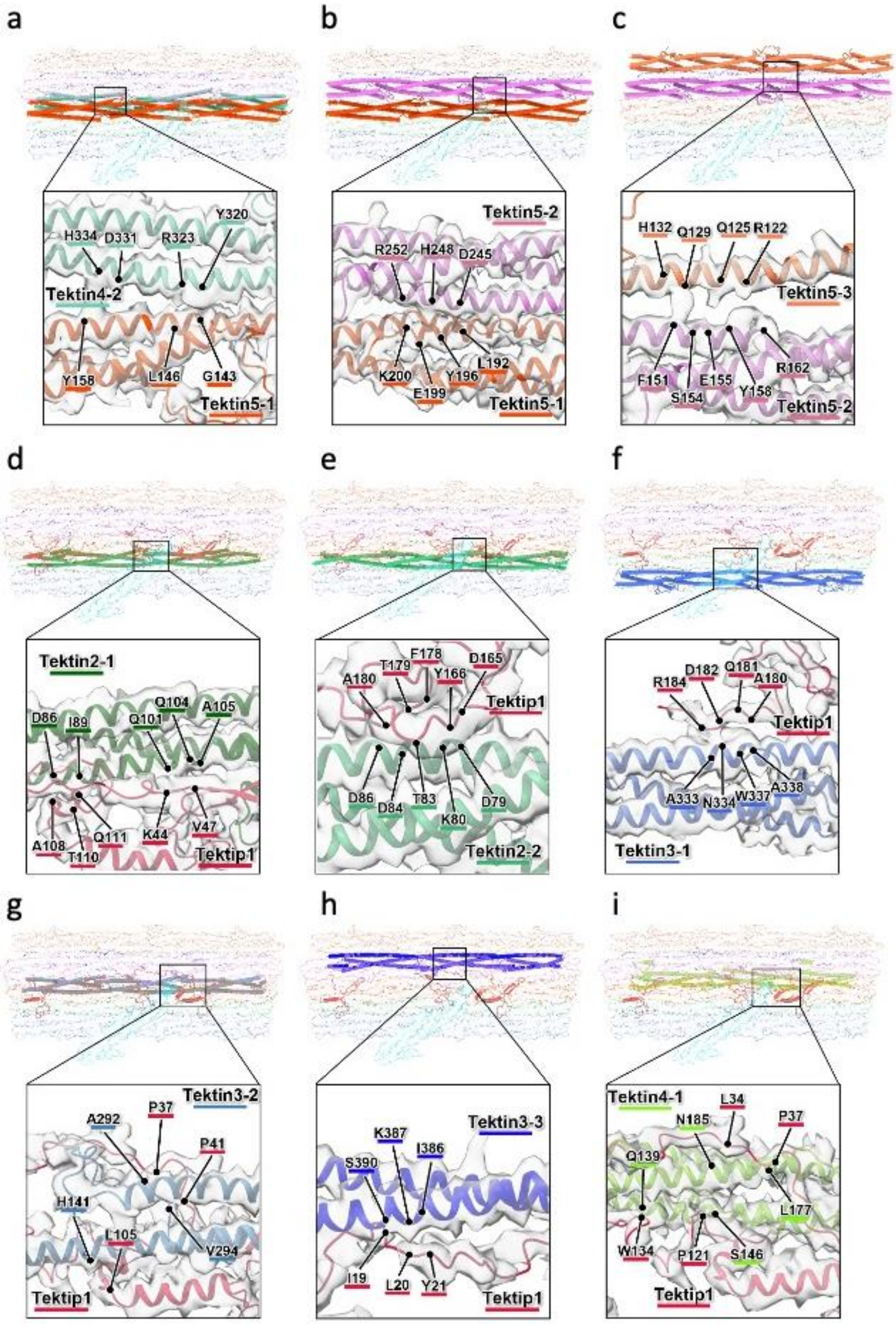

Supplementary Figure S17. Interactions among Tektin proteins within Tektin bundles. Interactions between Tektin4-2 and Tektin5-1 **(a)**, Tektin5-1 and Tektin5-2 **(b)**, Tektin5-2 and Tektin5-3 **(c)**, Tektin2-1 and Tektip1 **(d)**, Tektin2-2 and Tektip1 **(e)**, Tektin3-1 and Tektip1 **(f)**, Tektin3-2 and Tektip1 **(g)**, Tektin3-2 and Tektip1 **(h)**, and Tektin4-1 and Tektip1 **(i)**. The density map of DMT_{F16} is shown with the corresponding Tektin proteins fitted. The residues potentially involved in the interfacial interactions

are indicated as black dots and labelled accordingly. These residues were recognized using UCSF ChimeraX [1]. The color schemes of Tektin proteins are summarized in Supplementary Table S4.

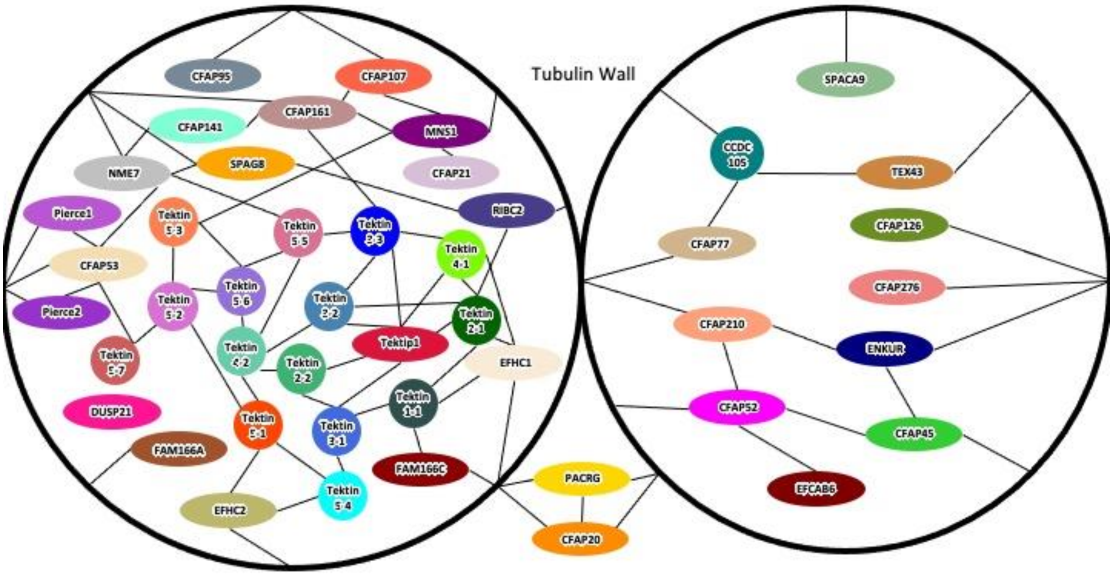

Supplementary Figure S18. Diagram of interaction pairs in DMT$_{F48}$ structure. The interacted proteins were recognized using default settings of UCSF ChimeraX's interface command [1]. The default probe radius for calculating each buried solvent-accessible surface is 1.4 Å. Interfaces with at least interface area of 300 Å^2 buried are counted as interfaces. Residues with at least interface area of 15 Å^2 are considered to be interface residues.

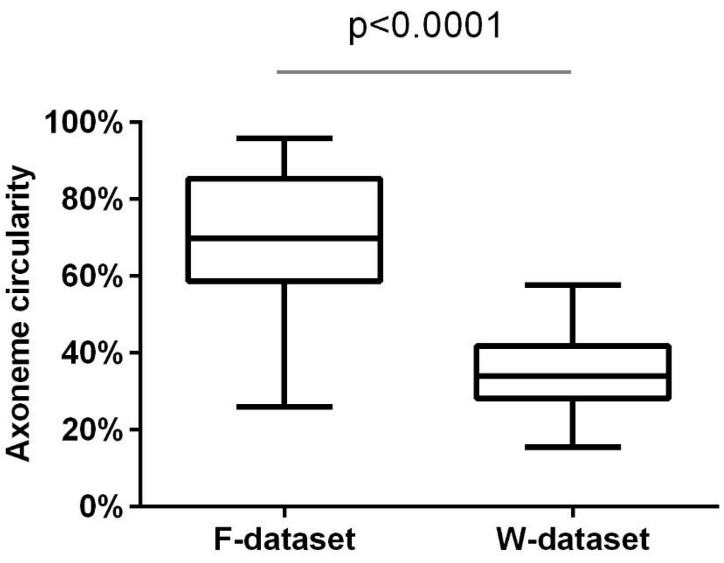

Supplementary Figure S19. Estimated axoneme circularities in F-dataset and W-dataset are presented in a Box-whisker Plot. The axonemes that contains 9 manually picked DMT were taken for calculation. This resulted in 108 axonemes for the F-dataset and 96 axonemes for the W-dataset. Subsequently, the DMT-center distance was calculated by measuring the distance between the axoneme center and each DMT. The axoneme circularity was estimated by determining the ratio of the smallest DMT-center distance to the largest DMT-center distance. The calculated axoneme circularity for the F-dataset is found to be 69.6% ± 17.2%, while for the W-dataset it is 34.7% ± 9.1%. Notably, the circularity has significant differences between the two datasets ($p<0.0001$).

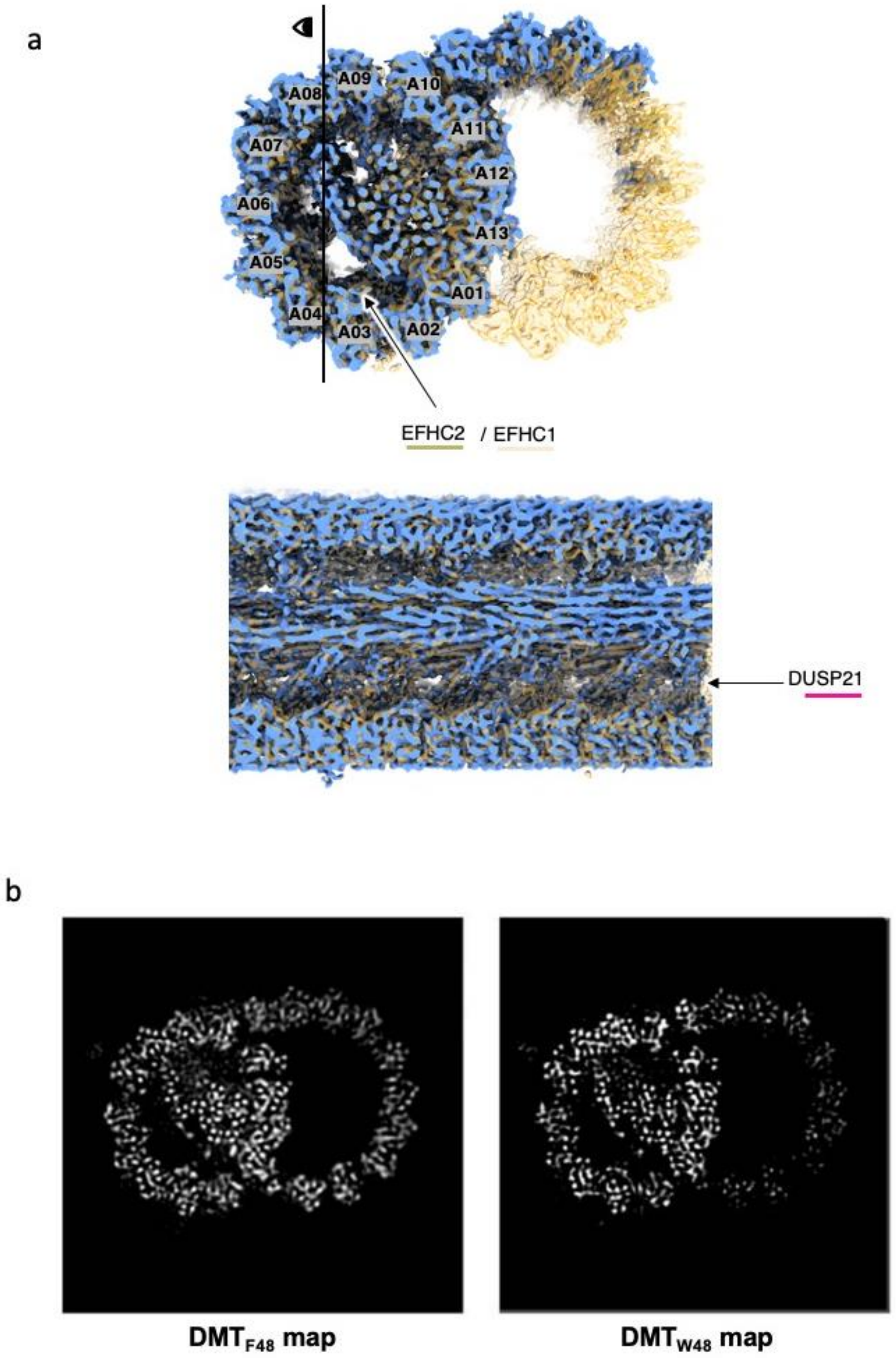

Supplementary Figure S20. Comparison of DMT_{W48} and DMT_{F48} maps. (a) The DMT_{W48} map (blue) is fitted into DMT_{F48} map (yellow), shown in transverse section and side views. **(b)** Grayscale images of DMT_{F48} and DMT_{W48} maps, shown in cross-section view.

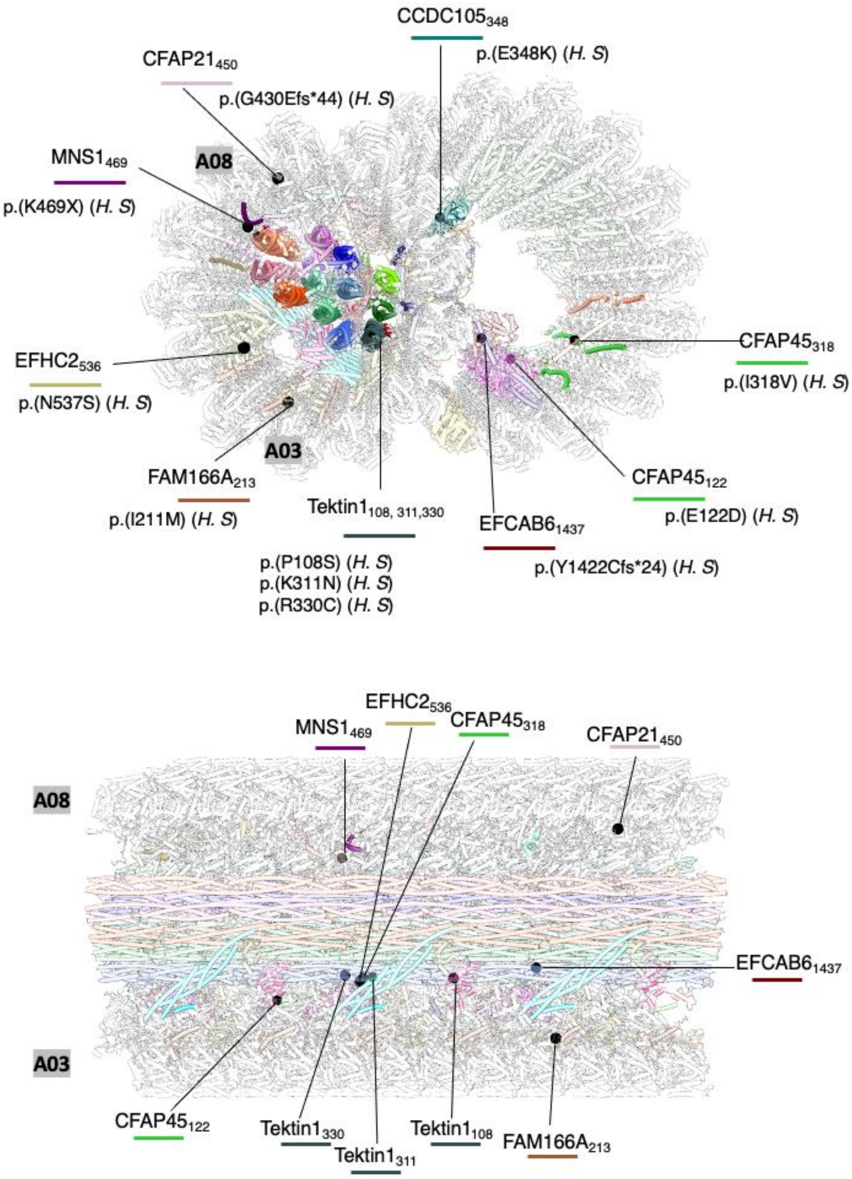

Supplementary Figure S21. Disease-associated mutations mapped on mouse sperm DMT structure. Several human disease-associated mutations[2] in sperm DMT MIPs are indicated and mapped onto the corresponding sites in mouse sperm DMT_{F48} structure. The structure is shown in both transverse section and side views. The color schemes for MIPs are summarized in Supplementary Table S4.

Supplementary Videos

Supplementary Video S1. Map and Model fitting quality of DMT_{F16}.

Supplementary Video S2. Map and Model fitting quality of DMT_{F48}.

Supplementary Video S3. Overall architecture of mouse sperm DMT in 48 nm repeats.

Supplementary Tables

Supplementary Table S1. Statistics of cryo-ET data collection, image processing and model building.

**Data acquisition**	
Microscope	Titan Krios G2
Voltage (kV)	300
Detector	Gatan K2
Energy filter	Gatan GIF Quantum, 20 eV
Mode	Super resolution
Pixel size (Å)	0.88
Stage tilting angle	-50° - 67° or -66° - 51°
Number of images	39 or 40
Exposure per image (e/Å²)	3 or 3.5
Exposure per tilt (e/Å²)	117 to 140
Defocus range (μm)	-1 - -4
Software	SerialEM

**Sub tomogram analysis**					
Software	RELION-3.1/Warp				
Data set	DMT$_{F16}$ map	DMT$_{F48}$ map	DMT$_{F96}$ map	DMT$_{W16}$ map	DMT$_{W48}$ map
EMDB ID entry (composite map)	EMD-35229 [#]	EMD-35230 [##]	EMD-35236 [###]	EMD-35237	EMD-35238
Number of tomograms	689	689	689	230	230
Number of particles	37018	17450	9389	12348	7580
Symmetry	C1	C1	C1	C1	C1
Resolution (Å)	4.5 ~ 6.5	6.5 ~ 7.5	7.7	7.9	8.6
Map pixel size (Å)	1.76	1.76	3.52	3.52	3.52

**Model building and refinement**		
Data set	DMT$_{F16}$	DMT$_{F48}$
PDB entry	8I7O	8I7R
Rmsd (bond) (Å)	0.005	0.004
Rmsd (angle) (°)	1.218	1.072
MolProbity score	2.41	2.34
No. of residues	67953	160892
No. of subunits	123	279
Ramachandran plot (%)		
Outliers	0.27	0.37
Allowed	7.33	6.37
Favoured	92.41	93.26
Rotamer outliers (%)	0.07	0.01

[#] This map was composed by the maps of A-tubule (EMD-35210, 4.5 Å) and B-tubule (EMD-35211, 6.5 Å).

[##] This map was composed by six maps, A1 part (EMD-35222, 6.5 Å), A2 part (EMD-35224, 6.6 Å), and A3 part (EMD-35225, 6.6 Å) of A-tubule, B1-part (EMD-35226, 7.5 Å), B2-part (EMD-35227, 7.5 Å), and B3-part (EMD-35228, 7.5 Å) of B-tubule.

This map was composed by two maps, half A (EMD-35231, 7.7 Å) and half B (EMD-35232, 7.7 Å).

Supplementary Table S2. The mouse MIPs in the structural model of DMT are all identified in the mass spectrometry assay.

Accession	Description	Score[#]	Found in Sample	Rank
P68372	Tubulin beta-4B	684.761	High	4
P05214	Tubulin alpha-3	466.724	High	21
G5E8A8	Tektin5	452.404	High	26
Q6P1E8	EFCAB6	308.565	High	72
Q6X6Z7	Tektin3	288.146	High	82
Q9D9T8	EFHC1	283.77	High	88
Q9D485	EFHC2	260.629	High	106
Q149S1	Tektin4	257.834	High	109
Q9D4K5	FAM166A	232.182	High	131
Q5F201	CFAP52	208.053	High	163
Q9DAJ2	Tektin1	207.888	High	164
Q922G7	Tektin2	195.799	High	185
Q8CDU5	CFAP21	189.021	High	198
Q9D9U9	CFAP45	185.006	High	205
Q9D4K7	CCDC105	139.41	High	299
A0JLY1	CFAP210	128.385	High	343
Q61884	MNS1	125.89	High	353
Q9D439	CFAP53	113.793	High	404
Q9QXL8	NME7	107.383	High	439
Q6SP97	ENKUR	101.476	High	474
Q7TPM5	SPACA9	86.071	High	576
Q9D4Q1	RIBC2	83.717	High	598
Q8BTU1	CFAP20	78.746	High	648
Q9DAK2	PACRG	77.46	High	663
A0A087WRI3	CFAP77	66.659	High	776
Q6P8Y0	CFAP161	53.633	High	946
Q3V0Q6	SPAG8	53.257	High	956
A6H6Q4	Tektip1	51.03	High	992
Q4KKZ1	CFAP107	50.851	High	996
Q9DAS2	FAM166C	43.839	High	1128
Q9DAD0	CFAP276	42.036	High	1163
Q9D9D8	DUSP21	41.535	High	1167
Q9CQC3	CFAP95	38.37	High	1240
Q5BN45	PIERCE1	36.029	High	1299
Q9D9I1	TEX43	33.599	High	1361
Q6P8X9	CFAP126	32.63	High	1383
V9GXK1	PIERCE2	18.579	High	1926
Q9D9D9	CFAP141	1.291	High	4274

[#]Score： Protein scores were calculated as the sum of the negative logarithms of the peptide values of the connected Phenol-soluble modulins.

Supplementary Table S3. FindMySequence scores of MIPs in DMT_{F16} map.

Protein	Score	Candidate	Gene name	Success (Y/N)
Tubulin_Alpha	1.30E-79	TBA1C	P68373	Y
	1.30E-79	TBA1A	P68369	
Tubulin_Beta	2.30E-63	TBB4B	P68372	Y
CCDC105	6.40E-37	CC105	Q9D4K7	Y
CFAP126	No Matches			N
CFAP20	1.30E-12	CFA20	Q8BTU1	Y
CFAP276	9.70E-03	M3K13	Q1HKZ5	N
CFAP52	1.90E-02	CFA52	Q5F201	Y
CFAP77	No Matches			N
DUSP21	6.40E-11	DUS21	Q9D9D8	Y
EFCAB6	1.80E-02	ZC4H2	Q68FG0	N
EFHC1	4.70E-21	EFHC1	Q9D9T8	Y
EFHC2	1.70E-43	EFHC2	Q9D485	Y
ENKUR	No Matches			N
FAM166A	No Matches			N
FAM166C	No Matches			N
PACRG	2.40E-05	PACRG	Q9DAK2	Y
SPACA9	No Matches			N
Tektin-1	3.30E-54	TEKT1	Q9DAJ2	Y
Tektin-2-1	1.30E-40	TEKT2	Q922G7	Y
Tektin-2-2	2.60E-39	TEKT2	Q922G7	Y
Tektin-3-1	1.90E-42	TEKT3	Q6X6Z7	Y
Tektin-3-2	4.90E-22	TEKT3	Q6X6Z7	Y
Tektin-3-3	1.30E-28	TEKT3	Q6X6Z7	Y
Tektin-4-1	3.10E-32	TEKT4	Q149S1	Y
Tektin-4-2	1.10E-43	TEKT4	Q149S1	Y
TEKTIP1	9.60E-03	TKTI1	A6H6Q4	Y
Tektin-5-1	8.10E-14	TEKT5	G5E8A8	Y
Tektin-5-2	1.60E-11	TEKT5	G5E8A8	Y
Tektin-5-3	No Matches			N
Tektin-5-4	No Matches			N

Supplementary Table S4. Top 3 of FindMySequence scores of MIPs in DMT_{F16} map.

Protein	Top1	Top2	Top3
Tubulin_Alpha	sp|P68373|TBA1C_MOUSE|E-value=8.60e-89	sp|P68369|TBA1A_MOUSE|E-value=9.90e-89	sp|P05214|TBA3_MOUSE|E-value=1.70e-88
Tubulin_Beta	sp|P68372|TBB4B_MOUSE|E-value=1.70e-66	sp|Q9D6F9|TBB4A_MOUSE|E-value=3.10e-65	sp|Q9CWF2|TBB2B_MOUSE|E-value=3.30e-65
Tektin1-1	sp|Q9DAJ2|TEKT1_MOUSE|E-value=2.80e-52	sp|Q149S1|TEKT4_MOUSE|E-value=5.10e-12	sp|Q6X6Z7|TEKT3_MOUSE|E-value=8.80e-05
Tektin2-1	sp|Q922G7|TEKT2_MOUSE|E-value=7.00e-31		
Tektin2-2	sp|Q922G7|TEKT2_MOUSE|E-value=6.10e-39		
Tektin3-1	sp|Q6X6Z7|TEKT3_MOUSE|E-value=1.80e-43	sp|G5E8A8|TEKT5_MOUSE|E-value=2.90e-14	sp|Q149S1|TEKT4_MOUSE|E-value=7.30e-04
Tektin3-2	sp|Q6X6Z7|TEKT3_MOUSE|E-value=3.80e-22	sp|G5E8A8|TEKT5_MOUSE|E-value=7.30e-06	
Tektin3-3	sp|Q6X6Z7|TEKT3_MOUSE|E-value=3.80e-28	sp|G5E8A8|TEKT5_MOUSE|E-value=1.20e-08	
Tektin4-1	sp|Q149S1|TEKT4_MOUSE|E-value=5.30e-31	sp|Q6X6Z7|TEKT3_MOUSE|E-value=3.70e-03	sp|G5E8A8|TEKT5_MOUSE|E-value=7.20e-03
Tektin4-2	sp|Q149S1|TEKT4_MOUSE|E-value=3.30e-43		
Tektip	sp|A6H6Q4|TKTI1_MOUSE|E-value=9.60e-03		
Tektin5-1	sp|G5E8A8|TEKT5_MOUSE|E-value=1.70e-16	sp|Q6X6Z7|TEKT3_MOUSE|E-value=4.80e-04	
Tektin5-2	sp|G5E8A8|TEKT5_MOUSE|E-value=3.60e-11	sp|O08599|STXB1_MOUSE|E-value=5.00e-02	
CCDC105	sp|Q9D4K7|CC105_MOUSE|E-value=4.40e-36		
CFAP20	sp|Q8BTU1|CFA20_MOUSE|E-value=1.30e-12		
CFAP52	sp|Q5F201|CFA52_MOUSE|E-value=2.60e-02		

DUSP21	sp|Q9D9D8|DUS21_MOUSE|E-value=1.60e-13	sp|Q8VE01|DUS18_MOUSE|E-value=8.00e-11	sp|Q9JLY7|DUS14_MOUSE|E-value=5.30e-05
EFHC1	sp|Q9D9T8|EFHC1_MOUSE|E-value=1.80e-20		
EFHC2	sp|Q9D485|EFHC2_MOUSE|E-value=1.70e-43		
PACRG	sp|Q9DAK2|PACRG_MOUSE|E-value=1.80e-04		

Supplementary Table S5. Color scheme of MIPs during structural analysis.

Name of MIP	Color scheme
Tektin1	dark slate gray
Tektin2-1	dark green
Tektin2-2	medium sea green
Tektin3-1	royal blue
Tektin3-2	steel blue
Tektin3-3	blue
Tektin4-1	lawn green
Tektin4-2	medium aquamarine
Tektin5-1	Orange red
Tektin5-2	orchid
Tektin5-3	coral
Tektin5-4	cyan
Tektin5-5	pale violet red
Tektin5-6	medium purple
Tektin5-7	indian red
Tektip1	crimson
EFHC1	antique white
EFHC2	dark khaki
FAM166A	sienna
FAM166C	dark red
DUSP21	deep pink
CCDC105	Teal
ENKUR	navy
TEX43	peru
CFAP276	light coral
CFAP126	olive drab
CFAP52	magenta
EFCAB6	maroon
CFAP77	tan
CFAP20	dark orange
PACRG	Gold
SPACA9	dark sea green
MNS1	purple
NME7	silver
CFAP53	wheat
CFAP161	rosy brown
RIBC2	dark slate blue
CFAP45	lime green
CCDC173	red
CFAP141	aquamarine
Pierce1	medium orchid
Pierce2	dark orchid
CFAP95	light slate gray
CFAP107	tomato
EFHB	medium spring green
SPAG8	orange
CFAP210	light salmon
CFAP21	thistle

Supplementary Table S6. Composition of sperm capacitive solution.

Reagent name	Mg/100ml	Vendor	Catalog number
**NaCl**	697.6	Sigma	S-5886
**KCl**	35.6	Sigma	P-5405
**$CaCl_2 \cdot 2H_2O$**	25.1	Sigma	C-7902
**Glucose (D+)**	100.0	Sigma	G-6152
**Soduim pyruvate**	5.5	Sigma	P-4562
**$MgSO_4 \cdot 7H_2O$**	29.3	Sigma	M-7774
**KH_2PO_4**	16.2	Sigma	P-5655
**$NaHCO_3$**	210.6	Sigma	S-5761
**Penicillin G K salt**	7.5	Sigma	P-4687
**Streptomycin sulfate**	5.0	Sigma	S-1277
**Methyl-beta-cyclodextrin**	98.3	Sigma	C-4555
**Polyvinylalcohol**	100.0	Sigma	P-8136

Supplementary Dataset

Supplementary Dataset S1. The mouse sperm proteome identified in mass spectrometry assay.

References

1 Pettersen, E. F. *et al.* UCSF ChimeraX: Structure visualization for researchers, educators, and developers. *Protein Sci* **30**, 70-82, doi:10.1002/pro.3943 (2021).

2 Zhou, L. *et al.* Structures of sperm flagellar doublet microtubules expand the genetic spectrum of male infertility. *Cell* **186**, 2897-2910 e2819, doi:10.1016/j.cell.2023.05.009 (2023).
